# Human Health, Economic and Environmental Assessment of Onsite Non-Potable Water Reuse Systems for a Large, Mixed-Use Urban Building

**DOI:** 10.3390/su12135459

**Published:** 2020-07-07

**Authors:** Sam Arden, Ben Morelli, Mary Schoen, Sarah Cashman, Michael Jahne, Xin (Cissy) Ma, Jay Garland

**Affiliations:** 1 Eastern Research Group, Lexington, MA 02421, USA;; 2 Soller Environmental, Berkeley, CA 94703, USA;; 3 United States Environmental Protection Agency, Center for Environmental Solutions and Emergency Response, Cincinnati, OH 45268, USA;

**Keywords:** non-potable reuse, decentralized treatment, life cycle assessment, life cycle cost assessment, quantitative microbial risk assessment, membrane bioreactor

## Abstract

Onsite non-potable reuse (NPR) is being increasingly considered as a viable option to address water scarcity and infrastructure challenges, particularly at the building scale. However, there are a range of possible treatment technologies, source water options, and treatment system sizes, each with its unique costs and benefits. While demonstration projects are proving that these systems can be technologically feasible and protective of public health, little guidance exists for identifying systems that balance public health protection with environmental and economic performance. This study uses quantitative microbial risk assessment, life cycle assessment and life cycle cost analysis to characterize the human health, environmental and economic aspects of onsite NPR systems. Treatment trains for both mixed wastewater and source-separated graywater were modeled using a core biological process—an aerobic membrane bioreactor (AeMBR), an anaerobic membrane bioreactor (AnMBR) or recirculating vertical flow wetland (RVFW)—and additional treatment and disinfection unit processes sufficient to meet current health-based NPR guidelines. Results show that the graywater AeMBR system designed to provide 100% of onsite non-potable demand results in the lowest impacts across most environmental and human health metrics considered but costs more than the mixed-wastewater version due to the need for a separate collection system. The use of multiple metrics also allows for identification of weaknesses in systems that lead to burden shifting. For example, although the RVFW process requires less energy than the AeMBR process, the RVFW system is more environmentally impactful and costly when considering the additional unit processes required to protect human health. Similarly, we show that incorporation of thermal recovery units to reduce hot water energy consumption can offset some environmental impacts but result in increases to others, including cumulative energy demand. Results demonstrate the need for additional data on the pathogen treatment performance of NPR systems to inform NPR health guidance.

## 1. Introduction

Water scarcity is a growing sustainability challenge facing many regions. The recent drafting of a National Water Reuse Action Plan in the U.S. [1] is evidence of a pressing need to address this challenge, as well as a reminder of work to be done to identify suitable reuse sources, end uses and treatment approaches.

Onsite non-potable water reuse (NPR) is one option to alleviate water scarcity challenges, particularly in large cities [2,3]. Onsite NPR systems capture and treat water generated within or surrounding a building, such as mixed wastewater or source-separated graywater, for reuse in toilet flushing, clothes washing and irrigation [3]. Besides alleviating water scarcity, onsite NPR can reduce the burden on existing drinking water and wastewater treatment systems, reduce building sewer fees, inspire community innovation and foster water system resilience through redundancy and source diversification [4].

Demonstration projects across the country are showing that onsite treatment of rainwater, stormwater, graywater and blackwater is technologically achievable and publicly acceptable [4–7]. Some cities are even requiring onsite reuse for certain new construction. San Francisco, for example, has an ordinance requiring new commercial, mixed-use or multi-family buildings over 250,000 square feet (23,226 square meters) to include onsite NPR [8]. Accordingly, a growing body of guidance literature has led to a risk-based framework for public health protection for onsite NPR [9–12], including pathogen log reduction targets (LRTs) to inform the selection of treatment configurations that achieve health risk benchmarks [12,13]. Still, onsite NPR systems are not widespread and guidance on proper technology selection and best design practices is lacking.

As onsite NPR becomes more common, there is an opportunity to incorporate additional measures of economic and environmental sustainability to inform the adoption of integrated urban water management (IUWM) principles [14,15]. A central tenant of IUWM is that potential options must be comprehensively evaluated in terms of economic, social and environmental aspects, requirements echoed in broader discussions of urban water system sustainability [16–18]. This facilitates greater transparency in the decision-making process [19,20] and helps identify problematic tradeoffs that can lead to negative consequences [21].

There are few examples of integrated assessments of the financial, social and environmental aspects of onsite NPR. Schoen et al. [22] used environmental, cost and quantitative microbial risk assessment (QMRA)-derived risk metrics to compare conventional and alternative water and sanitation systems, including onsite NPR. The alternative systems incorporating NPR had reduced environmental impacts, local human health impacts and cost compared to the conventional, centralized option, but their cost was highly variable compared to onsite sanitation options without NPR. However, only one NPR system was assessed and the technologies evaluated were designed for residential households, not large buildings.

Several studies have evaluated community-scale NPR systems using individual economic, environmental or human health metrics. The influence of treatment system capacity, degree of decentralization and treatment system technology has been evaluated using life cycle assessment (LCA) [23–25], LCA and life cycle cost assessment (LCCA) [26] and QMRA [10]. Both Cashman et al. [26] and Kavvada et al. [24,25] found that design flow or capacity economies of scale strongly influenced cost and environmental performance of decentralized membrane bioreactors (MBRs), with clear advantages for larger systems. However, they only evaluated larger, community-scale NPR systems, which have different distribution and collection requirements and pathogen risk profiles than single building systems. Hendrickson et al. [23] used LCA to compare a novel building-scale wetland treatment system for onsite NPR to a centralized conventional wastewater treatment plant and centralized NPR system. Although they found the wetland to be significantly less efficient than the conventional wastewater treatment plant, results showed the onsite wetland had energy consumption advantages when compared to centralized NPR, in line with suggestions that constructed wetlands can be a low-energy reuse option [27–29]. Schoen et al. [10] found that onsite MBR treatment of source-separated graywater or mixed wastewater at the large building scale could meet current human health benchmarks but that additional disinfection barriers would more robustly protect against protozoan pathogen risk, a conclusion echoed by a recent review of membrane treatment performance [30]. Similarly, the pathogen reduction performance of constructed wetlands is low relative to other biological processes [31], requiring still greater protection than MBRs.

To develop sound design and implementation guidance that can be widely adopted, it is critical that system economic, environmental and human health aspects be evaluated simultaneously to avoid burden shifting. For example, do the previously identified environmental benefits of decentralized MBRs [25,26] translate to single building applications, especially when coupled with more robust disinfection processes necessary for adequate human health protection (e.g., [10])? Likewise for low-energy treatment wetlands [23,28,31]. How does reduced potable water demand affect net system cost and environmental impacts? How are cost and environmental impacts affected by the unique requirements of additional, in-building collection and distribution piping for onsite NPR systems? When designed to the same standards, which system costs less? To our knowledge, such thorough analyses of onsite NPR systems have yet to be conducted.

To address these questions, we design onsite NPR treatment systems for a large, mixed-use building that meet defined human health risks guidelines [12,32,33] and comprehensively evaluate their health, cost and environmental aspects using QMRA, LCCA and LCA, respectively. Scenarios evaluated include different core biological treatment technologies used to treat either combined wastewater or source-separated graywater to meet the partial, full, or excess supply of building non-potable water demand. We focus on MBRs and treatment wetlands as core biological processes due to their prevalence in existing case studies and their demonstrated robust treatment performance at small scales. Specifically, we evaluate aerobic membrane bioreactors (AeMBRs), anaerobic membrane bioreactors (AnMBRs) and recirculating vertical flow wetlands (RVFWs). AeMBRs are a common, commercially viable treatment option (Hai et al., 2019). AnMBRs were investigated to explore the energy recovery potential of onsite wastewater treatment (Cashman et al., 2018). RVFWs were selected as a lower-energy, natural treatment option that relies on active recirculation to achieve a smaller land requirement than traditional constructed wetlands (Arden and Ma, 2018; Gross et al., 2007).

## 2. Methods

### 2.1. Treatment System Design

The design, operation and performance of wastewater treatment systems depend on influent water quality characteristics and the degree of treatment required to meet applicable standards or guidelines. For this study, source waters for reuse include mixed wastewater or source-separated graywater (bathroom faucets, laundry, showers and baths) with water quality characteristics specified in Table S1 [32]. Treatment systems include a core biological treatment process and any necessary pre-treatment, post-treatment or disinfection processes to meet applicable water quality guidelines for chemical/physical parameters [33,34] and human health protection [12,32].

Chemical/physical effluent guidelines were considered for total suspended solids (TSSs), biological oxygen demand (BOD) and the maintenance of a chlorine residual (Table S1).

**Figure 1.**
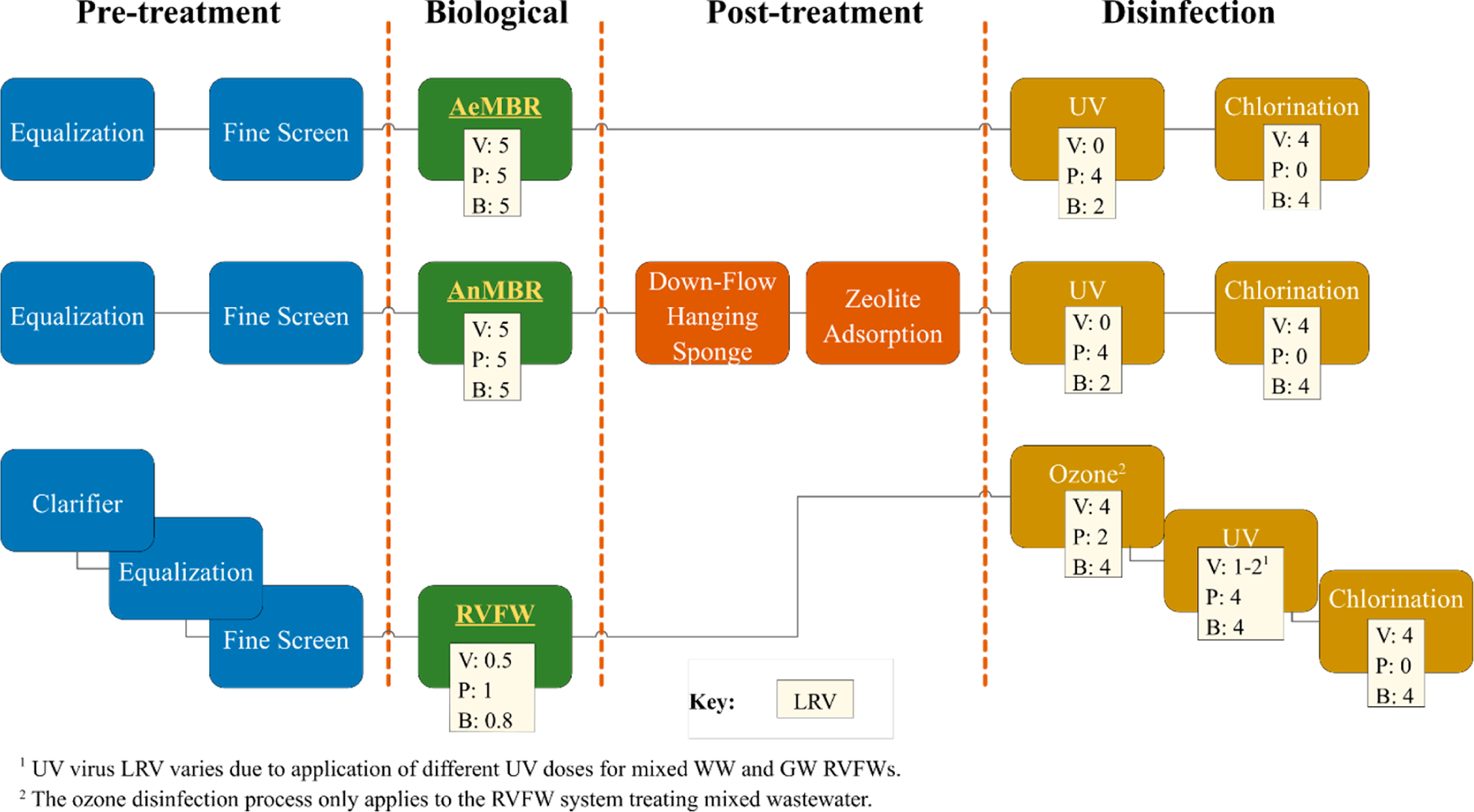
Onsite NPR treatment system unit processes. Reported LRVs apply to both mixed-wastewater and graywater treatment systems; for UV, different doses were used among the systems. AeMBR—aerobic membrane bioreactor, AnMBR—anaerobic membrane bioreactor, B—bacteria, LRV—log reduction value, P—protozoa, RVFW—recirculating vertical flow wetland, UV—ultraviolet, V—virus, and NPR—non-potable reuse.

Human health protection guidelines specify log reduction targets (LRTs) by source water type for specific reuse applications (Table 1). The LRTs correspond to a risk level of 1 in 10,000 infections per person per year (ppy) for mixed wastewater and graywater across three organism classes [12]. All treatment systems were designed to meet LRTs for indoor reuse, with the additional requirement that each pathogenic organism type have at least two barriers of protection to provide reliable treatment [12]. The lower half of Table 1 lists cumulative log reduction values (LRVs) achieved by each system using each process’ LRV outlined in the NPR guidance [12] (SI S1.1). Figure 1 and Table S3 show the LRVs assigned to individual unit processes and the corresponding disinfection dose.

### 2.2. Life Cycle Inventory Development

**Figure 2.**
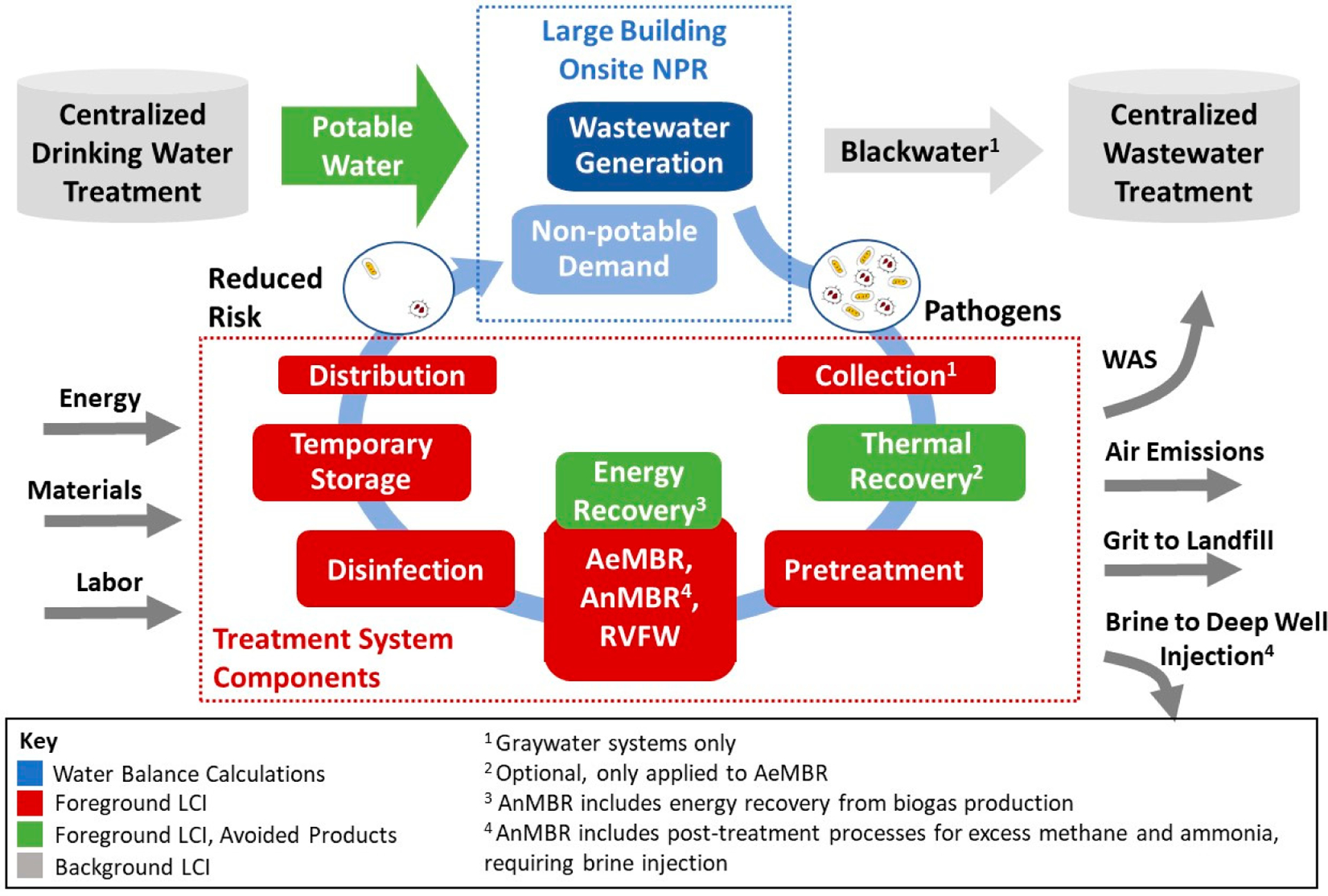
System diagram for the threeNPRwastewater treatment systems. AeMBR—aerobic membrane bioreactor, AnMBR—anaerobic membrane bioreactor, LCI—life cycle inventory, NPR—non-potable reuse, RVFW—recirculating vertical flow wetland, and WAS—waste activated sludge.

Life cycle inventories (LCIs) were developed for each treatment configuration. LCI data catalogue the material and energy inputs and emissions to nature that are associated with the operation of processes necessary to deliver the defined functional unit, which in this case includes the wastewater treatment facilities, building distribution, avoided products and other elements illustrated in Figure 2. SI Section S1 describes the development of LCI data for individual unit processes and Tables S9–S14 present the full LCIs of the foreground treatment systems. Table S21 provides the system component lifetimes that were assumed.

Each system includes pre-treatment, biological and disinfection unit processes. Centralized treatment of wastewater sludge and the untreated fraction of building wastewater is included in the LCI for all scenarios. For graywater systems, a separate collection system was modeled while all systems include a separate, non-potable distribution system. An optional thermal recovery system was also modeled to assess the benefit of recovering thermal energy from wastewater streams to offset onsite hot water heating demands. LCI data are representative of conditions in San Francisco, CA.

### 2.3. Water Use Scenarios

A range of treatment capacity scenarios were developed to evaluate the performance of the treatment systems under different conditions that may be encountered within a large, mixed-use building. Building characteristics were adapted from Morelli et al. [2]. The building stands 19 stories tall, has a total footprint of 20,000 square feet (1858 square meters) and houses 520 residents and 590 office workers. Wastewater generation and demand for NPR water are based on typical residential and commercial water use estimates [10,35,36], resulting in a building-wide non-potable demand of 0.013 million gallons per day (MGD) (49 cubic meters per day, or m^3^/d), graywater generation of 0.016 MGD (61 m^3^/d), and mixed-wastewater generation of 0.025 MGD (95 m^3^/d) (See SI Section S2 for additional discussion). The study’s functional unit is the delivery of NPR water for the whole building. Treated wastewater or graywater is distributed throughout the building, displacing the building’s need for potable water from the centralized treatment works. As designed, the treatment systems are transitional solutions, intended to maintain a connection with the local sanitary sewer, storm sewer and drinking water system. Blackwater, waste activated sludge and excess mixed wastewater and graywater not required to meet the building’s NPR demand are disposed of in the centralized sewer system.

In order to weigh the costs and benefits associated with different, plausible levels of NPR implementation, three treatment capacity scenarios are defined where onsite wastewater treatment provides 80% of non-potable demand (Partial Treatment Scenario), 100% of non-potable demand (Full Treatment Scenario) or 120% of non-potable demand (Excess Treatment Scenario) (Table S15). The Partial Treatment Scenario, where treatment systems are designed to satisfy only a portion of onsite non-potable demand, may be pursued where limited demand for reuse water exists or where limited funds are available for construction of the reuse system. The Full Treatment Scenario is intended to capture the most likely level of implementation. The Excess Treatment Scenario provides treatment for the largest volume of wastewater and may be pursued where additional future demand is expected.

### 2.4. QMRA

The human health impacts of exposure to pathogens from the selected NPR systems were predicted using QMRA [10,37]. The risk from ingestion of enteric pathogens in treated non-potable water was characterized as a probability of infection using viral and protozoan reference pathogens (the risk from bacteria was negligible in a preliminary screening analysis). The methodology, previously developed for NPR systems [10], accounts for natural variation in pathogen density in the source water and variation in treatment performance. AnMBR performance was not identified and thus assumed to be the same as that of AeMBR. This work also incorporates sudden treatment failure in line with what has been previously considered for potable reuse of water [38,39]. Note that this analysis did not rely on the guidance LRVs reported in Table 1; rather, it assessed the risk associated with the variable performance reported for these systems.

The annual probability of infection (P_inf,annual_) was calculated as:

(1) $${\text{P}}_{\text{inf},\text{annual}}=1-\prod_{n_{i}}\left[1-DR\left(V_{i*}\;10^{{\text{log}}_{10}\left(C\right)-\text{TP}}\right)\right]$$

Where

*DR*(…) is a dose-response function for the reference pathogen,

*V*_*i*_ is the volume of water ingested per day for use *i*,

*n*_*i*_ is the number of days of exposure over a year for use *i*,

*C* is the pathogen concentration in the untreated source water, and

TP is the treatment performance expressed as a log_10_ reduction in the total treatment processes

(e.g., TP = TP_MBR_ + TP_disinfection_).

The P_inf,annual_ in Equation (1) was calculated from a daily pathogen dose accumulated from non-potable indoor water use (the exposure routes are described in Section S3.1). Ten thousand Monte Carlo simulations in R version 3.3.1 [40] were implemented to capture the daily variation in pathogen concentration (described in Section S3.3) and treatment performance (LR), when available (described in Section S3.4). The volume ingested through inhalation and dermal contact, number of exposures and dose-response assessment parameters (described in Sections S3.1 and S3.2) were fixed at point values. The 95th percentile pathogen-specific annual risks were reported along with the combined risk (CP_inf_) across pathogens (p):

(2) $${\text{CP}}_{\text{inf}}\text{=}\;1-\underset{p}{\Pi }\left[{1-\text{P}}_{\text{inf,annual}}\right]$$

### 2.5. LCCA

LCCA was conducted using a net present value (NPV) method from the National Institute of Standards and Technology [41]. The NPV calculation depicted in Equation (3) was used to estimate the LCC of each system over a 30 year period. The NPV method allows one-time, periodic and annual costs to be assessed on a consistent basis that considers the time value of money using a conservative 5% real discount rate, representative of small-scale projects without access to the best interest rates. LCCA only considers cost escalation rates beyond the standard inflation rate for energy costs. Electricity and natural gas costs were escalated using factors specific to the California region [42].

(3) $$\text{Net}\;\;\text{Present}\;\text{Value=}\sum \left(\frac{{\text{Cost}}_{x}}{{\left(1+i\right)}^{x}}\right)$$

Where

NPV (2016 $) = net present value of all costs and revenues necessary to construct and operate the wastewater treatment facility,

*Cost*_*x*_ = cost in future year x,

*i* (%) = real discount rate, and

*x* = number of years in the future.

Total capital cost of individual treatment processes is the sum of unit process costs, direct costs and indirect costs. Unit process costs include equipment capital expenditures and installation cost. Direct costs represent costs required to integrate individual unit processes within the larger wastewater treatment system (Table S19). Indirect costs include additional expenditures such as professional services, profit and contingencies (Table S20). Indirect costs were estimated by applying indirect cost factors (Table S20) to the sum of unit process and direct costs plus interest during construction (Equation S1). An interest rate of 1.7% was used in the analysis, and represents the 2017 interest rate from California’s Clean Water State Revolving Fund [43].

Total annual cost is the sum of operation and maintenance labor, material, chemical and energy purchases. The cost of equipment replacement is included in material cost and considers the expected lifespan of individual system components (Table S21).

### 2.6. LCA

LCA is a methodology used to quantify environmental impacts of a defined product or process. LCA studies are carried out in four phases: (1) goal and scope definition, (2) inventory analysis, (3) impact assessment and (4) interpretation, as defined in ISO Standards 14040 and 14044 [44,45]. Details of phase 1 and 2 are described in Sections 2.1 and 2.2. The LCA includes eight environmental indicators as described in SI Section S5 and Table S22: acidification potential, cumulative energy demand, eutrophication potential, fossil fuel depletion, global warming potential, water use, particulate matter formation potential and smog formation potential. Selection of impact categories was based on categories present in U.S. EPA’s Tool for the Reduction and Assessment of Chemical and other environmental impacts, excluding toxicity-based impact categories due to a lack of data. Ozone depletion potential was excluded from the scope due to reduced relevance following implementation of the Montreal Protocol. Cumulative energy demand and water use provide useful summaries of inventory quantities.

### 2.7. Multiple Indicator Evaluation

To evaluate NPR options using multiple indicators, relative versions of each indicator are calculated. For all indicators except risk (*X*), the relative indicator increase is equal to the percent increase in the indicator value for scenario *i* relative to the minimum indicator result (Equation (4)). Minimum indicator results, representing options that produce the lowest (best) impact result, are equal to zero. For risk, the probability of infection for scenario *i* is divided by the health benchmark (10^−4^ ppy), such that a risk equivalent to the benchmark would be 100%, while a risk that is double the health benchmark would be 200%.

(4) $$\text{Relative}\;\text{indicator}\;\text{increase}=\frac{\left(X_{i}-X_{min}\right)}{\left|X_{min}\right|}$$

## 3. Results and Discussion

### 3.1. QMRA

**Figure 3.**
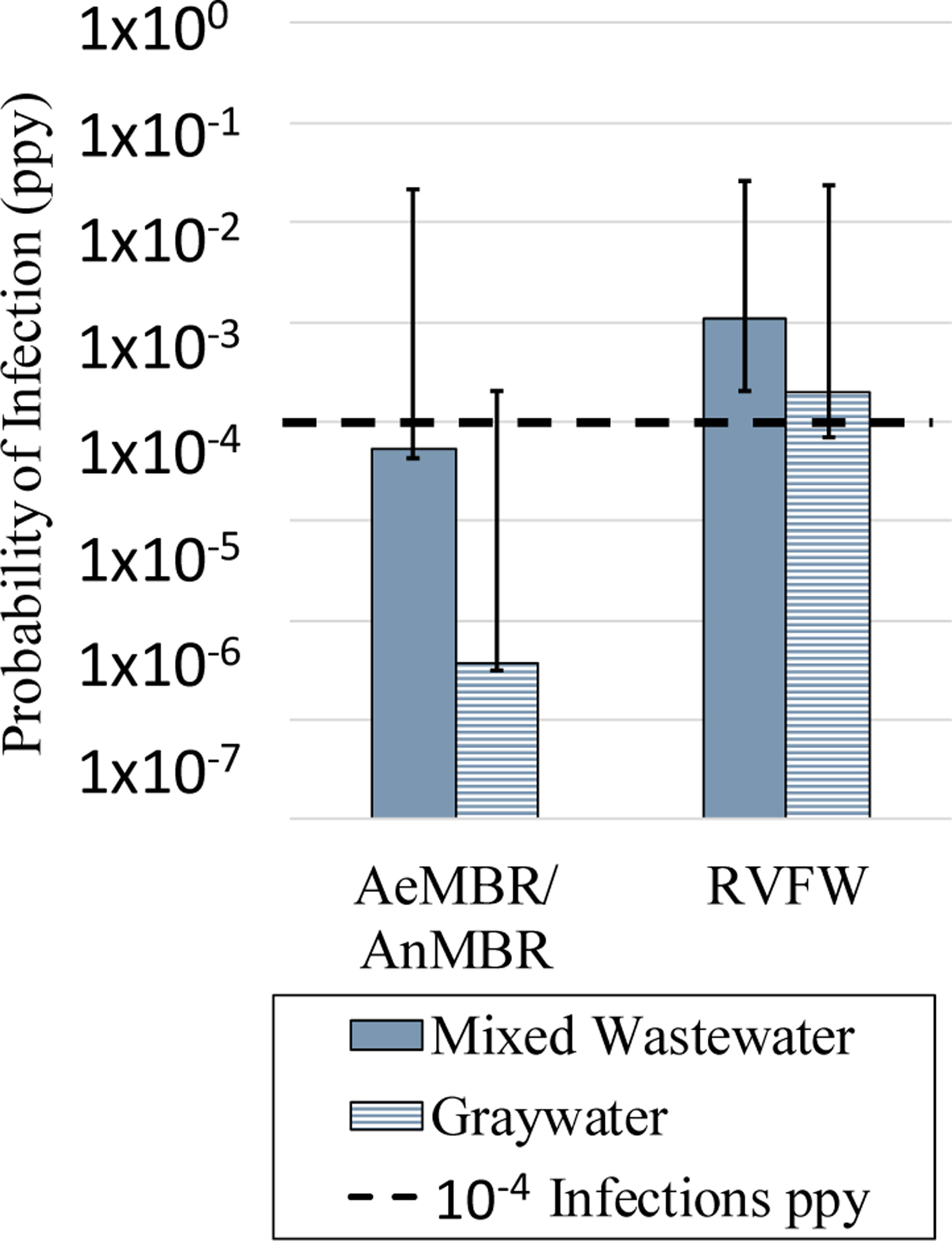
Comparison of the 95th percentile annual infection risk from NPR combined across pathogens for each treatment system. Bars represent risks calculated using the same dose-response functions used for the LRTs, i.e., the upper-bound dose response for *Cryptosporidium* and lower-bound dose response for *Norovirus*. The tails represent the risks calculated using the lower- and upper-bound dose responses. The AeMBR/AnMBR assumed the same treatment performance variability and thus have the same predicted risk. AeMBRs and AnMBRs share LRVs based on common use of ultrafiltration membranes.

Results in Figure 3 compare the 95th percentile annual probability of infection for the NPR treatment options. Results include treatment variability for AeMBR/AnMBR and ozone units and failure for the UV unit (see SI Section S3.4). Because the treatment units were selected to meet indoor NPR LRTs, the pathogen-specific annual risks in Table S23 (i.e., the risks numbered 1–4) were generally less than or just above 10^−4^ infections per person per year (ppy) using the dose-response assumptions consistent with the LRTs (numbers 2 and 3 in Table S23), with the exception of the *Cryptosporidium* spp. risk (number 2 in Table S23) for the wastewater RVFW. This relatively high risk is a result of the variable performance of ozone treatment. For comparison, the predicted risk for indoor use assuming the LRVs fell below the benchmark for all systems (Table S24). System risk using the upper-bound dose-response assumptions (the top tail in Figure 3 or number 4 in Table S23) is also high relative to the health benchmark. This is because the adopted *Norovirus* LRT (Table 1) was calculated assuming the lower-bound dose response [12].

Across system options, theAeMBR/AnMBRgraywater systems had superior performance based on combined pathogen risk. The total LRV was greater than the required LRT for graywater reuse systems, partially due to the applied criteria that each pathogen has at least two barriers of protection. The other options had risks more comparable to the infection risk benchmark (10^−4^ ppy). The mixed-wastewater AeMBR/AnMBR options ranked 2nd; the graywater RVFW ranked 3rd; and the mixed-wastewater RVFW had the highest combined risk. For the RVFW, the variation in ozone treatment performance had greater influence on the risk than the UV failure when modeled separately.

### 3.2. LCCA

**Figure 4.**
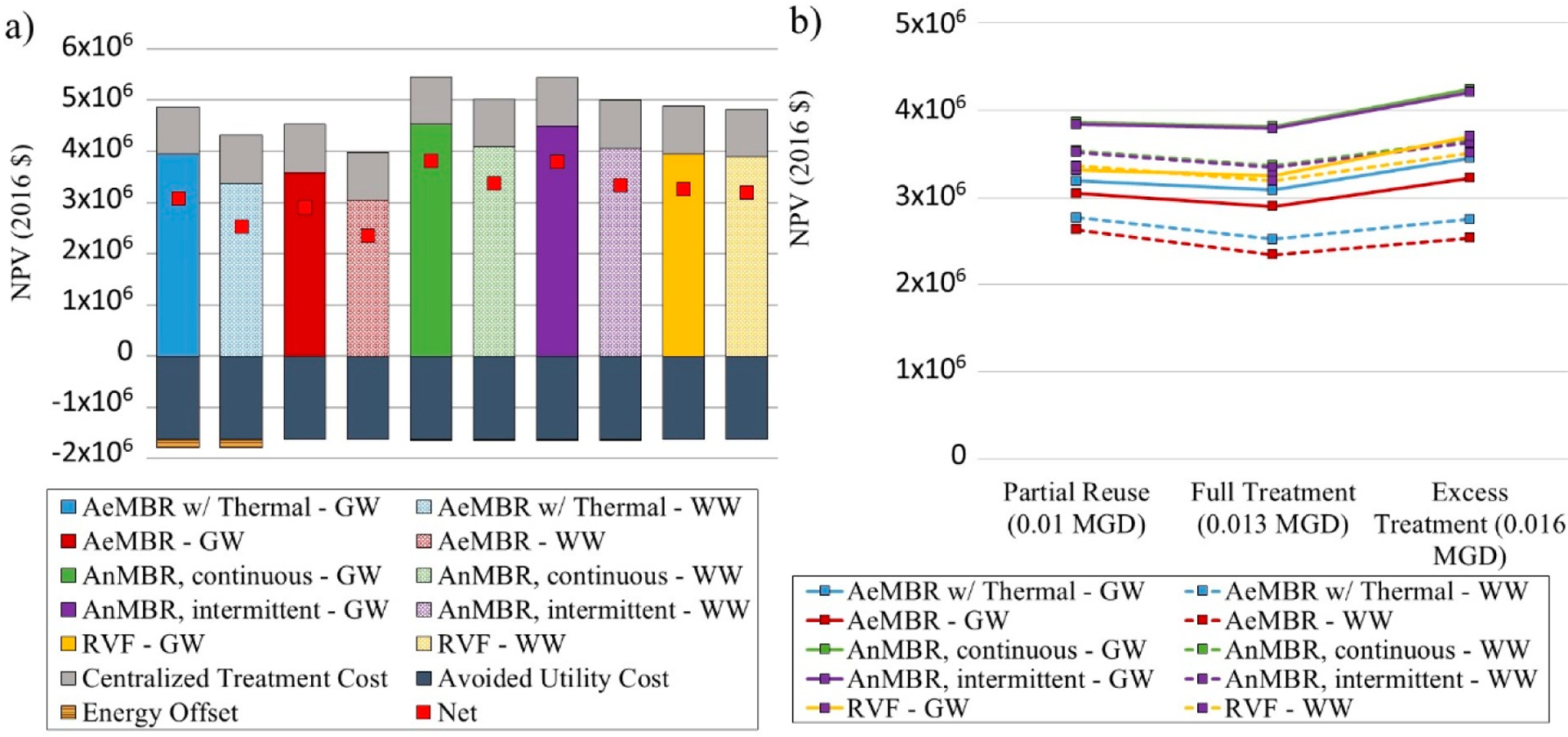
Life cycle cost assessment results of NPR systems: (**a**) shows results across systems for Scenario 2, where treatment capacity is equal to non-potable demand. Results include operation and infrastructure costs (positive), centralized wastewater treatment costs (positive), potable cost offsets (negative) and avoided energy cost (negative). Red squares indicate net costs; (**b**) shows NPV across Scenarios 1 through 3, where Scenario 2 costs (Full Treatment) correspond to net costs illustrated in Figure 4a. GW = graywater, WW= wastewater.

Figure 4 presents comparative LCCA results for building-scale mixed-wastewater and graywater treatment systems across several operational scenarios over a 30 year period. In Figure 4a, solid and patterned fill colors represent graywater and mixed-wastewater treatment systems, respectively. System NPV is lowest for the mixed-wastewater AeMBR without thermal recovery. Thermal recovery adds slightly to overall system cost when avoided natural gas costs are considered. Avoided costs (benefit) would be greater if electricity was the water heating energy source, as electricity is more expensive than natural gas per unit of delivered energy. The AnMBR, assuming either continuous or intermittent membrane sparging, is the most expensive treatment option. Energy savings associated with intermittent sparging do not considerably affect system NPV.

For both the AeMBR and AnMBR, total system NPV is less for the mixed-wastewater treatment systems, due primarily to the additional expense of a separate pipe network for graywater collection. Operational cost reductions such as reduced energy and chemical use for graywater systems are not sufficient to offset the additional piping cost. Many aspects of treatment system design such as tank size and membrane area are determined based not on wastewater strength but the volume of water treated, which leads to similar system costs for graywater and mixed-wastewater treatment systems.

Costs of the RVFW fall between the two membrane-based treatment systems and are similar for treatment of graywater and mixed wastewater. Due to low LRV for virus, protozoa and bacteria, the mixed-wastewater RVFW requires an additional ozone disinfection step, which balances the cost required for additional piping for the graywater system making costs more comparable across wastewater types.

Figure 4b shows net costs of each system across the three capacity scenarios. System costs decrease slightly in the Full Treatment Scenario due to economies of scale and maximization of utility cost savings. System costs are highest in the Excess Treatment Scenario owing to the larger treatment capacity and additional fees required for disposal of unused (treated) effluent into the combined sewer system.

### 3.3. LCA

**Figure 5.**
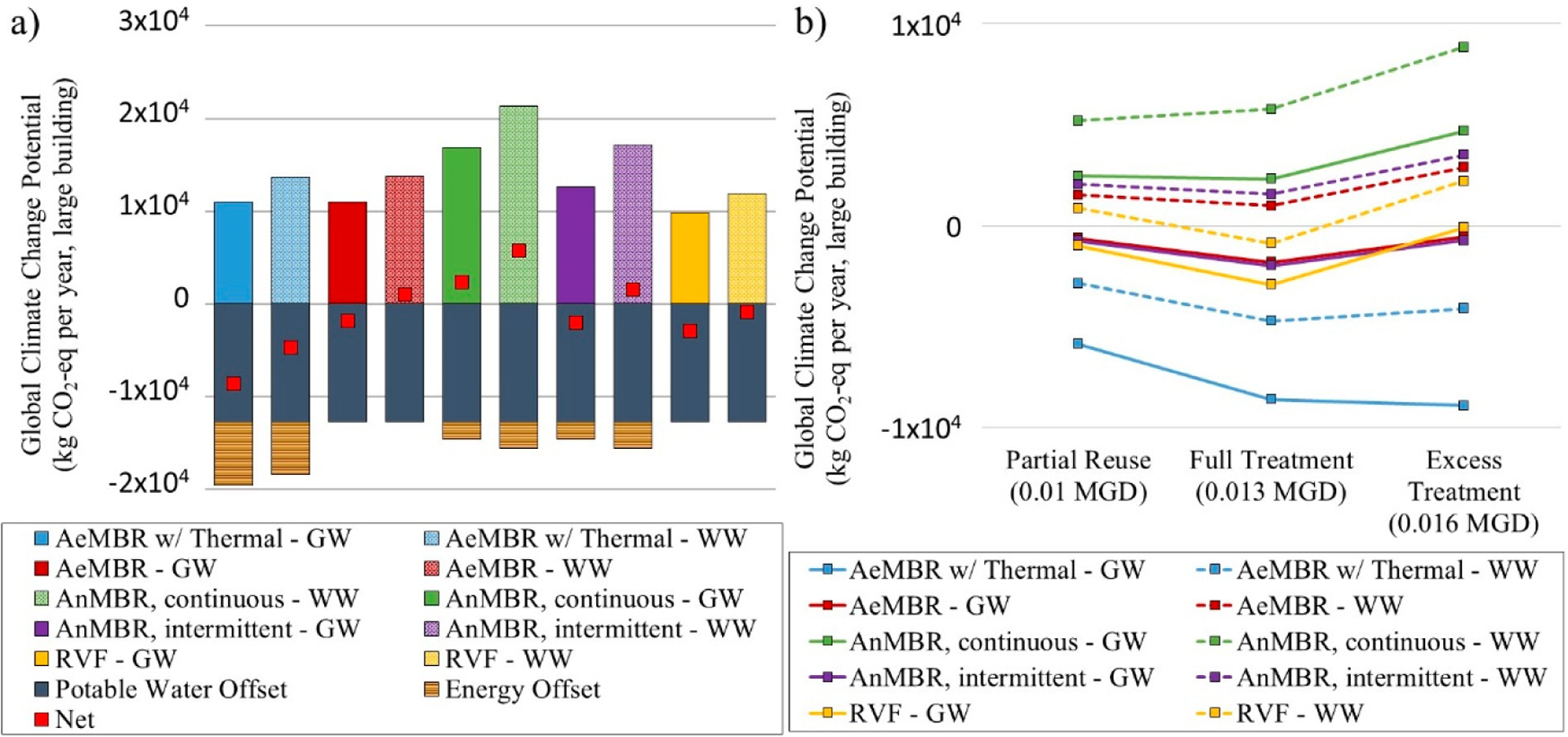
Global warming potential of NPR systems: (**a**) shows results across systems for Scenario 2, where treatment volume is equal to non-potable demand. Results include operation and infrastructure impacts (positive) and applicable avoided product credits (negative). Red squares indicate net impacts; (**b**) shows net impacts across Scenarios 1 through 3, where Scenario 2 (Full Treatment) corresponds to the net impacts illustrated in Figure 5a. GW = graywater and WW= wastewater.

Figure 5a displays GWP results for the Full Treatment Scenario where system treatment capacity equals non-potable demand (full LCA results are provided in Tables S21 and S22). Results include operation and infrastructure burdens as well as credits for reduced potable water demand. Avoided energy credits are shown for the thermal recovery unit combined with an AeMBR and biogas produced from the AnMBR, which both provide energy for building hot water heating. While the AnMBR recovers energy and produces less sludge than the AeMBR, these benefits are largely offset by additional post-treatment requirements and biogas sparging energy consumption. AnMBR results are more aligned with other treatment configurations when intermittent sparging can be employed, but this may impact overall system performance.

Net GWP impacts are lower for graywater systems than mixed-wastewater systems due to the reduced influent strength—mainly organic load—which generates less sludge, requires less aeration energy and results in fewer emissions of methane and nitrous oxide (Tables S9–S14, S21, S22). Graywater also has a higher influent temperature, which allows for more thermal energy recovery. For AnMBR systems, although mixed-wastewater versions can produce more biogas and achieve greater energy offsets than their graywater counterparts, this benefit is outweighed by greater treatment energy requirements and increased emissions resulting from treatment of higher-strength wastewater (Tables S11, S12, S25, S26).

Figure 5b illustrates the GWP impacts across the three capacity scenarios. Generally, GWP results are optimized when the treated water meets but does not exceed non-potable demand. The Partial Reuse Scenario has higher GWP impacts due to diseconomies of scale in material and energy requirements per volume of treated wastewater and a missed opportunity to capture potable offset credits. The Excess Treatment Scenario quantifies the penalty for treating more water than is required to meet NPR demand. The graywater thermal recovery scenario is an exception to this trend, where increased treatment system capacities always show a net GWP reduction due to the benefit of graywater thermal energy recovery.

### 3.4. Integrated Results

**Figure 6.**
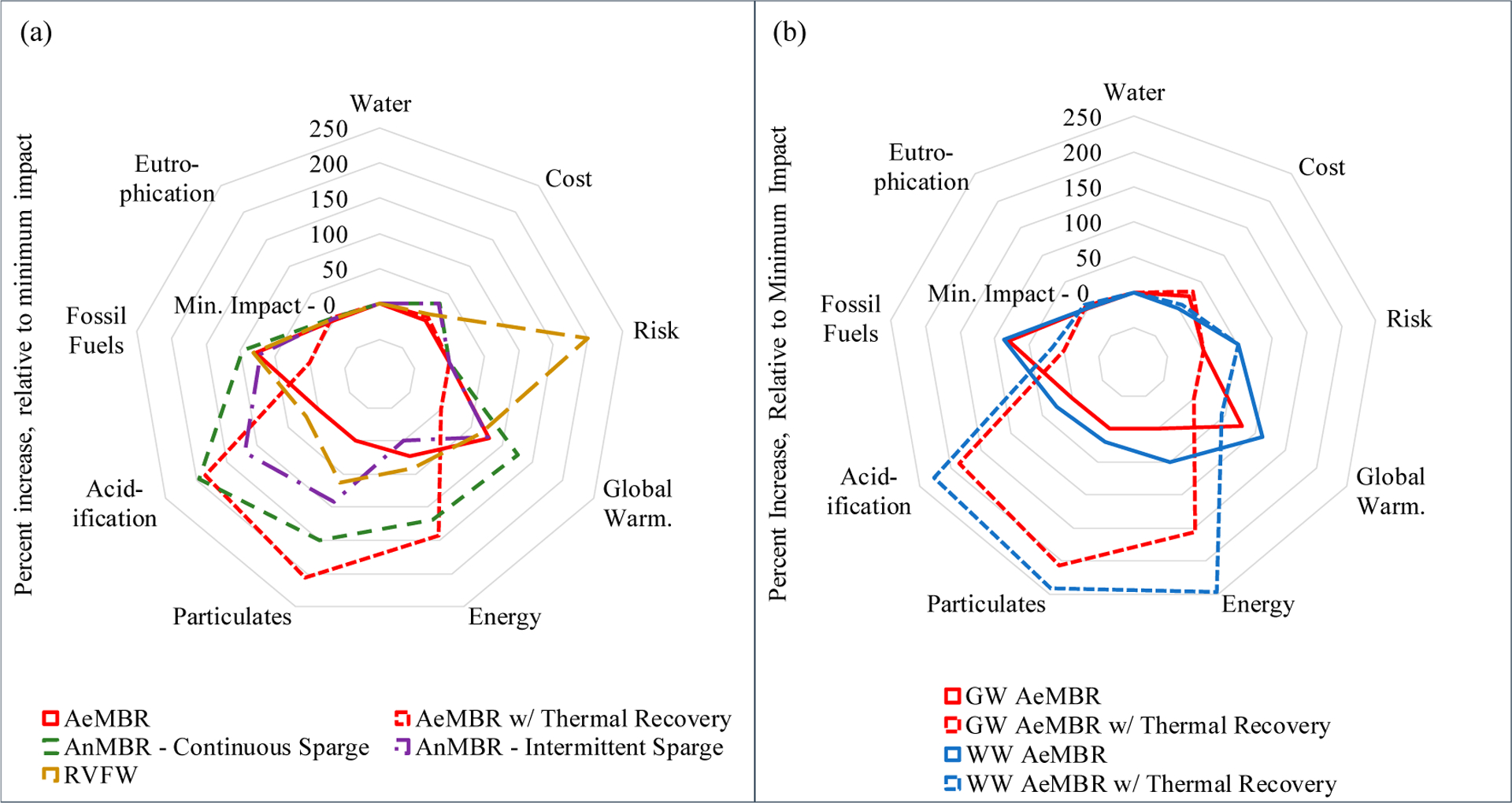
Summary of relative indicator results for seven environmental impact categories as well as cost and human health risk for (**a**) graywater treatment systems within the Full Treatment Scenario and (**b**) for the AeMBR treating either mixed wastewater or graywater. All results except risk are presented relative to the minimum result for that indicator in each figure, such that minimum impact equals zero. Risk results are presented relative to the health benchmark (10E-4 ppy), such that a risk equal to the health benchmark would be 100%. LCA metric translations: acidification—acidification potential, energy—cumulative energy demand, eutrophication—eutrophication potential, fossil fuel—fossil fuel depletion potential, global warming—global warming potential, particulates—particulate matter formation potential, and water—water use.

To evaluate the impact of treatment system type on overall performance, Figure 6a summarizes combined results for the Full Treatment Scenario graywater treatment systems. To evaluate the impact of source water selection on overall performance, Figure 6b summarizes combined results for the Full Treatment Scenario AeMBR systems treating either mixed wastewater or graywater. Results can be interpreted analogously to environmental footprint, where a smaller footprint is more desirable.

No one treatment system realizes the best performance across all indicators (Figure 6a). The RVFW system performs less favorably than the AeMBR due to the additional unit processes such as ozone treatment needed for pathogen reduction and still exceeds the health benchmark due to variable disinfection performance. The AeMBR system performs well across many of the metrics, including cost. AeMBR fossil fuel and global warming impacts can be decreased through incorporation of a thermal energy recovery unit offsetting natural gas hot water heating, but this water-to-water heat pump requires additional electricity which results in relatively higher impacts in other categories associated with electricity generation such as energy, acidification and particulates. AnMBR systems show higher overall impacts in many of the categories but impacts are reduced when intermittent biogas sparging is used. Summary LCA results for all mixed-wastewater and graywater treatment systems can be found in Tables S25 and S26.

In terms of source water type (Figure 6b), systems treating source-separated graywater outperform those treating mixed wastewater for all metrics except cost (Figure 4, Figure 6b), which shows little variability relative to differences in other categories.

## 4. Discussion

When reviewed in all metrics, results across human health, cost and environmental impact metrics show that some onsite NPR options perform consistently better than others, while exceptions provide insight into optimal configurations under specific contexts. The AeMBR system tends to perform best compared to the RVFW and AnMBR; however, its performance may be skewed based on its more advanced state of development. Unlike the AeMBR, AnMBR technology at this system size is not widely commercially available and the limited data available for LCI development come primarily from lab- and pilot-scale studies [2,46,47]. Results show that, aside from energy, even the mixed-wastewater AnMBR system with intermittent sparging (i.e., the design variation expected to perform best of all AnMBR variations) is outperformed by the AeMBR system (Figure 5, Figure 6). While the AnMBR system is able to produce enough biogas to decrease its energy impact relative to the AeMBR and RVFW systems, this benefit is offset by the costs associated with the additional unit processes required to address its limited ability to remove nutrients—mainly nitrogen—as well as the inclusion of metrics related to, but distinct from, cumulative energy demand such as global warming and particulates. In addition, due to requirements that the system maintain a specific internal temperature, thermal recovery is not suitable as a pre-treatment option, reducing the potential benefit that can be realized from the AnMBR system. The future optimization of AnMBR technology may provide different impact and cost outcomes.

The RVFW systems also do not perform as well as the AeMBR systems in terms of human health protection, cost and most LCA metrics. Although the RVFW has been shown to perform more consistently than other constructed wetland types in terms of organics and pathogen removal [31], its material and energy costs generally exceed those of the AeMBR systems when additional unit processes needed to meet effluent microbial risk guidelines are incorporated. For example, the RVFW biological AeMBR system. This finding highlights the challenge for wetlands to maintain their ‘low-energy’ competitive advantage when subject to more rigorous effluent guidelines, as is the case for San Francisco’s Living Machine wetland system which uses more than 2 kWh/m^3^ (Hendrickson et al., 2015). Conversely, passive wetlands not subject to human health-based effluent guidelines can require less than 0.1 kWh/m^3^ [28]. In addition, the variable pathogen reduction performance of the RVFW results in the system’s modeled risk exceeding the health benchmark despite being designed with a sufficiently high LRV.

In terms of source water type, systems treating source-separated graywater outperform those treating mixed wastewater for all metrics except cost (Figures 4 and 6b). Cost differences are not large, however; the difference between the NPV of graywater and wastewater AeMBRs for the Full Treatment Scenario is less than 20%, while differences in environmental benefits can be far greater. Specifically, graywater versions require less energy for aeration, generate fewer screenings and have lower emissions of methane and nitrous oxide. This is consistent with suggestions that, owing to its lower concentration of organics, pathogens and nutrients [48,49], treatment of source-separated graywater may be more efficient that mixed wastewater [17].

Incorporation of a thermal recovery unit to offset natural gas use associated with hot water heating demonstrates further tradeoffs. Although the thermal recovery unit reduces global warming and fossil fuel impacts of the AeMBR system with minimal additional cost, it results in much higher acidification and particulates impacts as well as overall higher energy use. These larger impacts result from the additional electricity required to run the heat exchanger (4.1 kWh/m^3^, Table S9) and depend on the emission factors associated with the fuel mix of the San Francisco power grid. These results will vary across the country depending on the local electrical grid mix as well as the type of hot water heater used (i.e., natural gas or electric) and should be explored further.

### 4.1. Limitations

QMRA results showed that when accounting for variable performance, annual risk of the RVFW wetland treatment systems exceeded the health benchmark. So, although the systems comply with the recommended LRVs, the actual risks may exceed the benchmark some of the time due to variation in treatment performance. To avoid this, the LRVs assigned to processes should be conservatively based on the worst or 5th percentile performance rather than average performance.

There remains outstanding uncertainty that was not included in the results but could change the predicted rankings with additional information. No LRVs were found for the RVFW, only for more rudimentary wetland systems. For the RVFW system to perform better than the MBR system, the RVFW LRVs for viruses and protozoa would need to exceed 3.0 for graywater treatment without ozone or increase to 1 and 2.5, respectively, for wastewater treatment with ozone. In addition, due to a lack of monitoring data in distributed systems, performance data for the MBR and ozone units and the UV failure frequency were primarily derived from centralized municipal treatment systems. If distributed operation and maintenance is less rigorous than for centralized treatment, then the annual health risk from exposure to pathogens in treated non-potable water could increase.

Operational cost data for all systems were based on centralized municipal treatment systems as no single design or operational standard currently exists (Tables S19 and S20, also see [2] for additional discussion). Further research is needed to demonstrate what level of monitoring and operational control is necessary to provide consistent, protective treatment performance.

A formal uncertainty assessment was not carried out for either the cost or environmental analysis, and appropriate caution should be used when interpreting the presented results. For example, both the RVFW and AnMBR treatment systems are innovative technologies in their infancy and less widely implemented than AeMBRs, which are commercially available. The novelty of the former contributes both to wide uncertainty around the underlying inventory values and the potential for future improvements. Moreover, economies of scale can have a significant effect on the cost and environmental performance of these systems [25,26]. Additional research is needed to explore the effects of building size and occupancy on the environmental and economic cost of these systems.

The underlying inventory data are specific to the San Francisco region, most notably utility fees, electrical grid mix, and centralized wastewater and drinking water treatment infrastructure. While most aspects of building and treatment systems are applicable to other regions, environmental impacts and costs associated with regionally specific factors do have a substantive effect on the absolute magnitude of results. For example, tradeoffs shown for thermal recovery incorporation will vary with grid mix, and differences may be more pronounced if the unit is used to offset an electric hot water heater rather than natural gas. Still, the influence of local conditions is expected to be less pronounced when focusing on comparative performance rather than the overall magnitude of individual metrics.

### 4.2. Decision Analysis

The objective of this study is to show how integrated metrics can comprehensively characterize onsite NPR options for large buildings, not to make a single recommendation. To be incorporated into a decision-making process, local stakeholder values must be applied to the provided, incommensurable metrics. For example, these results could be used as input to a multiple-criteria decision analysis (MCDA), where each metric is assigned a weight and a final ranking of options is made based on the context-specific combination of weighted metrics [50,51]. Cole et al. [20] provide a useful framework for MCDA in an IUWM context, as illustrated through a stakeholder-driven process to implement a dual water supply system.

## 5. Conclusions

This study presents the results of an integrated assessment that examines onsite NPR options for a large mixed-use building. Human health risk, cost and several environmental impact indicators were used to evaluate the effects of treatment system type, source water selection and treatment system capacity system performance. No one option performed best, though several general conclusions can be drawn from those options and approaches that performed consistently well. Although the context of the study was based on an actual building, the relative conclusions are intended to be broadly applicable.

According to health, cost and environmental indicators, AeMBRs tended to perform better than AnMBRs and RVFWs, as the latter are both challenged by the need for additional pre-treatment (RVFW), post-treatment (AnMBR) and disinfection processes (RVFW). Cost was the only indicator by which mixed-wastewater versions of each treatment technology had a comparable advantage over their graywater counterparts due to the cost of additional piping required for source-separated graywater collection. In terms of environmental indicators, graywater versions outperformed mixed-wastewater versions, largely due to lower energy inputs and reduced emissions associated with treating lower-strength wastewater. Thermal recovery from graywater to offset natural gas use associated with onsite hot water heating improves system GWP and FDP, but at the expense of large increases in AP, PMFP and CED. Last, displacement of potable water consumption is a key determinant of total system cost and environmental performance. Systems designed to meet, but not exceed, onsite non-potable demand performed best.

## Supplementary Material

Supp Info

## Figures and Tables

**Table 1.** Log Reduction Targets for Indoor and Unrestricted Irrigation Use and Log Reduction Values of Mixed-Wastewater and Graywater Treatment Systems.

Reuse Type	Wastewater Type	Log Reduction Target^a^		
		Enteric Viruses	Parasitic Protozoa	Enteric Bacteria
Indoor Use	Mixed wastewater	8.5	7	6
	Graywater	6	4.5	3.5
Unrestricted Irrigation	Mixed wastewater	8	7	6
	Graywater	5.5	4.5	3.5
Treatment System	Wastewater Type	Log Reduction Values^b^		
		Enteric Viruses	Parasitic Protozoa	Enteric Bacteria
AeMBR and AnMBR^c^	Mixed wastewater	9	9	11
	Graywater	9	9	11
RVFW	Mixed wastewater	9.5	7	12.8
	Graywater	6.5	5	8.8

a Log reduction targets reproduced from [12];

b Log reduction values assigned using the approach outlined in SI Section S1.1;

c Log reduction values for AeMBRs and AnMBRs are based on the use of ultrafiltration membranes. AeMBR—aerobic membrane bioreactor, AnMBR—anaerobic membrane bioreactor, and RVFW—recirculating vertical flow wetland.
