# Supplementary material for: Human Health, Economic and Environmental Assessment of Onsite Non-Potable Water Reuse Systems for a Large, Mixed-Use Urban Building
Source: Sustainability. Author manuscript; Available in PMC 2021 Jul 7. (PMC7490829; doi:10.3390/su12135459)
Supplement: Supp Info [file NIHMS1622225-supplement-Supp_Info.pdf]

Supplemental Material: Human health, economic and environmental assessment of onsite non-potable reuse systems for a large, mixed use urban building

Sam Arden[1], Ben Morelli[1], Mary Schoen[2], Sarah Cashman[1], Michael Jahne[3], Cissy Ma[3*], Jay Garland[3]

[1]Eastern Research Group, Lexington, Massachusetts USA
[2]Soller Environmental, Berkeley, California, USA
[3]United States Environmental Protection Agency, Center for Environmental Solutions and Emergency Response, Cincinnati, Ohio USA

S1. Treatment System Design and Inventory Development

Life cycle inventories (LCI) were developed for each treatment configuration based on the designs necessary to achieve the desired effluent water quality discussed in the main document. This supplementary information section provides additional detail as to the specific design details of individual treatment system unit process. It is intended to support the overview provided in Section 2.1 of the main text. Table S1 [1] provides the physical/chemical water quality parameters assumed for mixed wastewater and source-separated graywater, as well as applicable effluent quality guidelines. The remainder of the section discusses the selection process and specific design details for individual unit processes.

Table S1. Wastewater Influent Characteristics and Target Effluent Quality for Unrestricted Urban Reuse

Water Quality Characteristics		*Influent Values*		*Target Effluent Quality*
		Mixed WW	Graywater	Both
Characteristic	Unit	Medium Strength	Low Pollutant Load with Laundry	Effluent Quality for Unrestricted Urban Use
Suspended Solids	mg/L	220	94	<5
Volatile Solids	%	80	47	-
$cBOD_5$	mg/L	200	170	-
BOD_5	mg/L	240	190	<10
COD	mg/L	510	330	-
TKN	mg N/L	35	8.5	-
Ammonia	mg N/L	20	1.9	-
Nitrite	mg N/L	-	-	-
Nitrate	mg N/L	-	0.64	-
Total Phosphorus	mg P/L	5.6	1.1	-
Chlorine Residual	mg/L	-	-	0.5-2.5

Table Acronyms: BOD – biochemical oxygen demand, cBOD- carbonaceous biochemical oxygen demand, COD – chemical oxygen demand

S1.1 Unit Process Selection to Achieve LRTs

Disinfection processes were specified for each of the wastewater treatment systems based on log reduction targets (LRTs) intended to achieve a risk level of 1 in 10,000 infections per person per year (ppy)

considering several reuse applications. Log reduction values (LRVs) vary based on organism type, disinfection method, and applied dose as specified in Table S2. Biological processes also provide some level of treatment, which was taken into account when selecting disinfection unit processes so that the total (additive) LRT could be achieved. Table S3 shows LRVs assigned to individual biological and disinfection processes included in the study systems, and the corresponding disinfection dose.

Both MBR treatment processes were assigned a LRV of five for each pathogen class, which is conservative based on the LRV of six or more reported by [2]. Based on a lack of available data for the RVFW specifically, it was assigned LRVs for wetlands from Sharvelle et al. (2017), varying between 0.5 and 1 depending on organism type.

Most systems only require chlorine and ultraviolet (UV) disinfection processes to meet LRTs for non-potable reuse. Chlorination is legally required for all non-potable reuse systems, in order to maintain a free chlorine residual of 1 mg/L [2].

The RVFW treating mixed wastewater requires a third disinfection process, ozone, to meet the LRTs for viruses and protozoa.

Table S2. Log Reduction Values for Biological and Disinfection Processes (Sharvelle et al., 2017).

		**Enteric Viruses**	**Parasitic Protozoa**	**Enteric Bacteria**	**Units**
**Membrane Bioreactor**[a]	Log Reduction	5	5	5	log
**Wetland**		0.5	1	0.8	log
**Free Chlorine**	1 Log_{10}	n/a	2000-2600	0.4-0.6	mg-min/L
	2 Log_{10}	1.5-1.8	n/a	0.8-1.2	mg-min/L
	3 Log_{10}	2.2-2.6	n/a	1.2-1.8	mg-min/L
	4 Log_{10}	3-3.5	n/a	1.6-2.4	mg-min/L
**Ozone**	1 Log_{10}	n/a	4-4.5	0.005-0.01	mg-min/L
	2 Log_{10}	0.25-0.3	8-8.5	0.01-0.02	mg-min/L
	3 Log_{10}	0.35-0.45	12-13	0.02-0.03	mg-min/L
	4 Log_{10}	0.5-0.6	n/a	0.03-0.04	mg-min/L
**UV Radiation**	1 Log_{10}	50-60	2-3	10-15	mJ/cm^2
	2 Log_{10}	90-110	5-6	20-30	mJ/cm^2
	3 Log_{10}	140-150	11-12	30-45	mJ/cm^2
	4 Log_{10}	180-200	20-25	40-60	mJ/cm^2

[a] Log reduction values for AeMBRs and AnMBRs are based on the use of ultrafiltration membranes.

Table S3. Log Reduction Values of Selected Wastewater Treatment Processes.

MBR - mixed WW	Virus	Protozoa	Bacteria	Dose	Dose Units
Technology	LRV	LRV	LRV		
Membrane bioreactor	5	5	5	n/a	n/a
Ozone	-	-	-	-	-
UV	0	4	2	30	mJ/cm^2
Chlorination	4	0	4	32	mg-min/L
Total System LRV	9	9	11		
MBR - graywater	Virus	Protozoa	Bacteria	Dose	Dose Units
Technology	LRV	LRV	LRV		
Membrane bioreactor	5	5	5	n/a	n/a
Ozone	-	-	-	-	-
UV	0	4	2	30	mJ/cm^2
Chlorination	4	0	4	32	mg-min/L
Total System LRV	9	9	11		
RVFW - mixed WW	Virus	Protozoa	Bacteria	Dose	Dose Units
Technology	LRV	LRV	LRV		
RVFW	0.5	1	0.8	n/a	n/a
Ozone	4	2	4	8.3	mg-min/L
UV	1	4	4	55	mJ/cm^2
Chlorination	4	0	4	32	mg-min/L
Total System LRV	9.5	7	12.8		
RVFW - graywater	Virus	Protozoa	Bacteria	Dose	Dose Units
Technology	LRV	LRV	LRV		
RVFW	0.5	1	0.8	n/a	n/a
Ozone	-	-	-	-	mg-min/L
UV	2	4	4	95	mJ/cm^2
Chlorination	4	0	4	32	mg-min/L
Total System LRV	6.5	5	8.8		

S1.2 *Pre-treatment*

Each of the three treatment systems utilize a fine screen and equalization chamber for pre-treatment. The fine screen removes large particles and debris from influent that could damage or impede operation of the biological treatment units. Screenings are disposed of in a municipal solid waste landfill. A slant plate clarifier also precedes the RVFW to prevent unnecessary clogging of the media beds. Equalization chambers were sized to dampen fluctuation in hourly wastewater generation within the building. The LCI of these three processes includes electricity use and basic infrastructure materials (steel, concrete, and piping).

S1.3 *Aerobic membrane bioreactor*

The AeMBR combines a continuously-stirred aerobic reactor with a submerged membrane filter for solids separation. Solids are pumped from the reactor and disposed of in the sanitary sewer, where they are treated with the rest of the municipal waste stream.

Table S4 presents basic design values for the mixed wastewater and graywater AeMBR treatment processes. LCI electricity consumption accounts for aeration energy demand to provide both biological process aeration and membrane cleaning, permeate pumping, sludge pumping, and miscellaneous additional uses. The membrane is made out of polyvinyl fluoride and was sized based on the wastewater flowrate and the design membrane flux of 20 liters per m^2 per hour (LMH). The analysis assumes a membrane lifespan of ten years [3]. Inputs of concrete and steel for tank construction were estimated based on the presented unit dimensions. Sodium hypochlorite is used for membrane cleaning and was estimated assuming that 950 liters of 12.5% NaOCl are used annually per 1,650 m^2 of membrane area [4].

Table S4. AeMBR Design Values

**Parameter**	**Mixed Wastewater**	**Graywater**	**Units**
Solids Retention Time[a]	15		days
Hydraulic Retention Time[a]	5		hours
Mixed Liquor Suspended Solids[b]	12,000	11,000	mg/L
Dissolved Oxygen Setpoint	2		mg O_2/L
Membrane flux	20		LMH
Backflush flux[c]	40		LMH
Membrane area, operation	200	130	m^2
Membrane area, total	300	190	m^2
Tank depth, operational	2.7	2.7	m
Tank length	3.3	2.1	m
Tank width[d]	1.1	1.1	m
Tank volume, operational	20	13	m^3
Physical cleaning interval[e]	10		minutes
Physical cleaning duration[e]	45		seconds
Chemical cleaning interval[e]	84		hours

[a] [5]

[b] Output of GPS-X model, dependent on selected SRT.

[c] Twice membrane flux [5].

[d] Tank width refers to individual tank. AeMBR consists of three parallel tanks.

[e] [6]

Table Acronyms: LMH – liters per m^2 per hour

The LCI includes process greenhouse gas (GHG) emissions of methane and nitrous oxide developed using the IPCC Guidelines of National Inventories [7]. Methane and nitrous oxide emissions were estimated based on the quantity of BOD and total kjeldahl nitrogen (TKN) entering the AeMBR treatment process, respectively. GPS-X™ was used to estimate BOD and TKN concentrations influent to the AeMBR.

S1.4 Anaerobic membrane bioreactor

The AnMBR is a psychrophilic treatment process intended to operate at ambient temperatures, eliminating heat demand typical of many anaerobic processes, and producing methane as a beneficial by-product that is assumed to be used as an alternative heat source for the building's hot water supply. The treatment process includes an anaerobic continuously stirred tank reactor (CSTR) and additional tanks to house the submerged membranes. Neither nitrogen or phosphorus are removed from wastewater in anaerobic reactors [8]. Therefore, downflow-hanging sponge (DHS) and zeolite adsorption post-treatment processes are necessary to ensure that treated effluent meets the criteria for unrestricted urban reuse. The DHS reactors recover or destroy methane dissolved in AnMBR permeate and have the additional benefit of removing 55% and 73% of COD and BOD remaining the wastewater. A zeolite adsorption system is used to remove ammonium from the wastewater to allow establishment of a free chlorine residual without excessive sodium NaOCl demand.

The AnMBR is a psychrophilic treatment process intended to operate at ambient temperatures, eliminating heat demand typical of many anaerobic processes, and producing methane as a beneficial by-product. The assumed temperature of influent mixed wastewater and graywater is 23^{0}C and 30^{0}C, respectively. Graywater temperature was calculated as the median of values reported in literature reviews of graywater treatment and reuse studies [9–12]. The mixed wastewater temperature is typical of medium strength domestic wastewater [13]. The treatment process includes an anaerobic continuously stirred tank reactor (CSTR) and additional tanks to house the submerged membranes.

Table S5 lists basic design and operational parameters of the mixed wastewater and graywater AnMBRs. The AnMBR has a 60 day solids retention time (SRT). Dimensions of the CSTR were estimated based on the influent flowrate and a hydraulic retention time (HRT) of eight hours. Membrane area and material requirements were determined based on wastewater flowrate and the design membrane flux of 7.5 LMH.

Inputs of concrete and steel needed for tank construction were estimated based on the presented unit dimensions. Electricity consumption of the AnMBR includes sludge pumping, operation of CSTR mixers, permeate pumping, and biogas recirculation (i.e., sparging) for membrane cleaning. The baseline scenario models continuous biogas sparging to ensure consistent performance, while intermittent sparging is assessed in a sensitivity analysis [14]. Sodium hypochlorite is used for periodic chemical cleaning of the membrane, with the same chemical requirement as discussed for the AeMBR.

Table S5. AnMBR Design Values

System Component	Parameter	Mixed Wastewater	Graywater	Units
Anaerobic Reactor	Solids retention time[a]	60		days
	Hydraulic retention time	8		hours
	Mixed liquor suspended solids	12,000		mg/L
	COD/BOD removal	90%		of influent concentration
	Tank diameter	4	3.5	m
	Tank height	4.8	4	m

Table S5. AnMBR Design Values

System Component	Parameter	Mixed Wastewater	Graywater	Units
	Mixing power	0.84	0.53	HP
	Biogas production	14	6.3	m^3/day
	Biogas recirculation[a]	120	76	m^3/hour
Membrane Tank	Flux[a]	7.5		LMH
	Membrane area, operational	530	340	m^2
	Membrane area, total	790	500	m^2
	Tank depth, per train	3.7		m
	Tank length, per train[c]	0.73	0.47	m
	Tank width, per train[c]	2.7		m

[a] [15,16]

Table Acronyms: BOD – biochemical oxygen demand, COD – chemical oxygen demand, LMH – liters per m^2 per hour

Anaerobic processes generate methane which is trapped under the floating cover. The LCA quantifies the benefit of avoiding natural gas consumption, assuming that generated biogas is used as an alternative heat source for the building's hot water supply. Biogas production was estimated as a function of COD removal, assuming that 90% of influent COD is removed [15,17,18]. Methane is produced at a rate of 0.25 and 0.26 m^3 CH_4 per kg of COD removed in the 23^{0}C and 30^{0}C reactors, respectively [19]. Five percent of produced methane was assumed to be lost through gaps in the floating cover, contributing process GHG emissions [20]. Neither nitrogen or phosphorus are removed from wastewater in anaerobic reactors [8]. All influent TKN was assumed to be released in the form of ammonia. Membrane processes produce effluent with less than 2 mg/L of total suspended solids [21].

Downflow-hanging sponge (DHS) and zeolite adsorption post-treatment processes are necessary to ensure that treated effluent meets the criteria for unrestricted urban reuse. The DHS reactors recover or destroy methane dissolved in AnMBR permeate and have the additional benefit of removing 55% and 73% of COD and BOD remaining the wastewater. Performance of the two-stage DHS system was based on the research of [22]. Methane removed from permeate in the stage-one reactor is recovered, contributing additional avoided natural gas benefits. Overall, the DHS reactor recovers or destroys 99.3% of permeate methane. Methane remaining in the treated wastewater following the DHS reactor was assumed to be off-gassed contributing further process GHG emissions. Electricity consumption of the DHS reactors includes wastewater pumping and blower operation. Steel, concrete, and piping material requirements were estimated based on unit dimensions.

A zeolite adsorption system is used to remove ammonium from the wastewater to allow establishment of a free chlorine residual without excessive sodium NaOCl demand. Ammonium adsorbs to zeolite in a packed bed reactor, which is then flushed with sodium chloride (NaCl) facilitating reuse of zeolite media. The resulting nitrogen rich brine solution is disposed of via deepwater injection, requiring 1.8 kWh of electricity per cubic meter of injected brine. Deng et al. [23] indicates that such a system should be able to remove greater than 95% of influent ammonium. The system was designed assuming an initial zeolite adsorption capacity of 3.1 mg NH_4-N per gram of zeolite media, which maintains sufficient adsorption capacity throughout nine regeneration cycles. Average adsorption capacity across the nine regeneration cycles is 2.4 mg NH_4-N per gram zeolite. Sodium hydroxide (NaOH) is also included in the LCI to raise the pH of the regeneration fluid, considerably reducing the NaCl requirement [23].

S1.5 Recirculating vertical flow wetland

The RVFW is a wetland based treatment process that uses active and continuous wastewater recirculation [24,25] to minimize land area requirements, making the process suitable for urban environments. Clarified wastewater is circulated over the surface of wetland planters. Wastewater filters downward through a 0.6 meter thick media bed consisting of crushed limestone and gravel. The media bed is suspended 0.5 meters above a concrete collection tank, into which wastewater falls, facilitating aeration. From the collection tank, water is recirculated to the surface.

Wastewater recirculation was determined based on results of a pilot-scale system (Gross et al. 2007), which reports that 8-12 hours of recirculation were sufficient to reach steady-state BOD and TSS removal when recirculating 300 liters of wastewater over one square meter of wetland area. This corresponds to treatment of 0.6 cubic meters of wastewater per square of wetland area per day. Sklarz et al. [25] identified an optimal recirculation rate of 1.5 meters (depth) per hour over the entire wetland surface. On average the system was assumed to remove 94% and 98% of influent TSS and BOD, respectively [24–27]

Process GHG emissions of nitrous oxide were estimated based on an emission factor of 0.006 kg N_2O/m^2 wetland area per year [28]. Methane emissions were estimated using the IPCC method and the average methane correction factor specified for vertical subsurface flow constructed wetlands [7].

Pump electricity requirements were estimated using the identified recirculation rate and estimated headloss in the distribution piping. Steel grating is included in the wetland design to suspend the media bed above the concrete collection basin. High-density polyethylene piping is used for wastewater distribution.

S1.6 Disinfection Processes

All treatment systems use chlorination and UV disinfection processes while the RVFW treating mixed wastewater requires a third disinfection process. Ozone was selected for its effectiveness against both viral and protozoan pathogens and the desire for a second barrier of protection against protozoa.

Liquid sodium hypochlorite (NaOCl) is used as the chemical disinfectant. Development of the result LCI value considers instantaneous chlorine demand due to ammonia and total organic carbon (TOC) present in the treated wastewater as well as chlorine decay in the contact basin. Electricity consumption was estimated for operation of the peristaltic pump.

The UV disinfectant dose is based on delivered UV intensity considering nominal UV intensity, transmittance of the quartz sleeve, bulb age, and bulb output in the UV spectrum. Commercially available Sanitron® UV units were specified based on the required delivered dose necessary to meet LRTs. Manufacturer specifications provide estimates of electricity consumption [29].

Ozone is produced from liquid oxygen in a Primozone® GM series ozone generator. Manufacturer specifications were used to develop LCI quantities for liquid oxygen and electricity consumption [30]. Ozone is injected into the effluent stream at the beginning of a three basin contact chamber. Instantaneous ozone demand is satisfied in the first chamber and is assessed on the basis of residual COD. Average ozone concentration in the second two chambers is used as the basis of effective ozone dose, considering ozone decay. Ozone decay was assessed assuming first-order decay and an average ozone half-life of 20 minutes [31].

S1.7 Thermal recovery

The analysis also looked at scenarios where the AeMBR treatment process was paired with a thermal recovery system. A heat pump is used to extract thermal energy from influent wastewater, transferring that thermal energy to the building’s hot water system, and avoiding natural gas consumption. Wastewater and graywater enter a heat pump at 23^0C and 30^0C, respectively. A coefficient of performance (COP) is used to express the efficiency of the heat recovery process. Combined COPs, which consider both compressor and pump operation, of 2.5 and 2.6 were used for mixed wastewater and graywater treatment systems,

respectively [32]. Estimates of obtainable thermal power are based on the temperature difference between wastewater as it enters and exits the heat pump, which was estimated to be 4.2^{0}C and 4.3^{0}C for mixed wastewater and graywater treatment systems, respectively [32]. Total thermal recovery is the sum of obtainable thermal power plus the fraction of compressor power transferred to the working fluid less internal loss in the heat pump [33]. The thermal recovery LCI also includes electricity consumption of the pump and compressor, fugitive emissions of the R-134a refrigerant used in the heat pump [34], and avoided natural gas consumption.

S1.8 Collection and Distribution Systems

Distribution of the recycled water for NPR requires its own piping system. Graywater recycling also requires a separate collection system. The collection and distribution systems were modelled as polyvinyl chloride (PVC) for the main vertical and zone risers, while crosslinked polyethylene (PEX) was modelled for in-unit main and distribution piping [35]. Recycled water was assumed to displace potable water treatment and distribution, with a 20% loss rate of water modelled during centralized treatment and distribution [36]. Displaced energy requirements from potable water distribution were based on the national median value from the review of literature sources in Xue et al. [37]. Although other background inventories were based on conditions reflective of the San Francisco region, the city's unique water supply system is gravity fed and distribution energy is anomalously low [38]. Net pumping energy for delivery of onsite recycled water was calculated as the difference between gross onsite pumping requirements and energy for potable water vertical pumping after taking into account the distribution pressure of the potable water supply [39].

S1.9 System Scaling

To adapt LCIs to different treatment capacities in a way that maintained original design characteristics and isolated the effects of treatment capacity on system cost and environmental impact, LCI components of individual unit processes were scaled in ways that maintained original design specifications (e.g., HRT, oxygen transfer rates, chemical dosage rates, etc.) but updated applicable dimensional line items (e.g., concrete, steel, energy, etc.). Tables S6 through S8 provide detail as to how individual LCI components of AeMBR, AnMBR and RVF systems were scaled. Impacts and cost of thermal recovery units were held constant per unit of flow. Final LCIs are provided in Tables S9 through S14.

Table S6. Scaling approach for AeMBR LCI components

Unit Process	Parts Description	Unit	Constant/ Variable[a]	Scaling Approach
Fine Screen	Electricity	kWh	Variable	Energy use equation from [40]
Fine Screen	Screening Disposal	kg	Constant	Constant fraction of flow
Fine Screen	Steel	kg	Constant	Constant screen area per unit of flow
Equalization	Concrete	m^3	Variable	Basin volume scaled to maintain HRT and depth to area ratio.
Equalization	Steel	kg	Variable	Basin volume scaled to maintain HRT and depth to area ratio.
Equalization	Electricity	kWh	Variable	Pumping energy varied as function of flow, adherence to original design equations
AeMBR	Concrete	m^3	Variable	Basin volume scaled to maintain HRT and depth to area ratio.
AeMBR	Steel	kg	Variable	Basin volume scaled to maintain HRT and depth to area ratio.
AeMBR	Polyvinyl Fluoride	kg	Constant	Constant membrane area per unit of flow
AeMBR	Sodium Hypochlorite	kg	Constant	Constant dose rate
AeMBR	Electricity	kwh	Variable	Pumping energy varied as function of flow, adherence to original design equations
AeMBR	Methane	kg	Constant	Constant fraction of flow
AeMBR	N2O	kg	Constant	Constant fraction of flow
AeMBR	Sludge	m^3	Constant	Constant fraction of flow
UV	Electricity	kWh	Constant	Constant dose rate
UV	Steel	kg	Constant	Number of units increased/decreased to maintain constant UV dose
Chlorination	Concrete	m^3	Variable	Basin volume scaled to maintain HRT and depth to area ratio.
Chlorination	Steel	kg	Variable	Basin volume scaled to maintain HRT and depth to area ratio.
Chlorination	Electricity	kwh	Constant	Constant electricity per unit of flow
Chlorination	Sodium Hypochlorite	kg	Constant	Constant dose rate
Storage	HDPE	kg	Constant	Number of units increased/decreased to maintain constant storage capacity

[a] Constant refers to line items that are constant per unit of flow treated. Examples include chemical dose rates, such as 3 mg of NaOCl per liter of water treated.

211

Table S7. Scaling approach for AnMBR LCI components

Unit Process	Parts Description	Unit	Constant/ Variable[a]	Scaling Approach
Fine Screen	Electricity	kWh	Variable	Energy use equation from [40]
Fine Screen	Screening Disposal	kg	Constant	Constant fraction of flow
Fine Screen	Steel	kg	Constant	Constant screen area per unit of flow
Equalization	Concrete	m^3	Variable	Basin volume scaled to maintain HRT and depth to area ratio.
Equalization	Steel	kg	Variable	Basin volume scaled to maintain HRT and depth to area ratio.
Equalization	Electricity	kWh	Variable	Pumping energy varied as function of flow, adherence to original design equations
AnMBR	Concrete	m^3	Variable	Basin volume scaled to maintain HRT and depth to area ratio.
AnMBR	Steel	kg	Variable	Basin volume scaled to maintain HRT and depth to area ratio.
AnMBR	HDPE	kg	Variable	Updated to account for new basin dimensions
AnMBR	Polyvinyl Fluoride	kg	Constant	Constant membrane area per unit of flow
AnMBR	Sodium Hypochlorite	kg	Constant	Constant dose rate
AnMBR	Electricity	kwh	Variable	Pumping energy varied as function of flow, adherence to original design equations
AnMBR	Methane	kg	Constant	Constant fraction of flow
AnMBR	Sludge	m^3	Constant	Constant fraction of flow
AnMBR	Biogas Recovery	m^3	Constant	Constant fraction of flow
DHS	Electricity	kWh	Constant	Constant per unit of flow
DHS	Methane	kg	Constant	Constant per unit of flow
DHS	Natural Gas	m^3	Constant	Constant per unit of flow
DHS	Concrete	m^3	Variable	Basin volume scaled to maintain HRT and depth to area ratio.
DHS	Steel	kg	Variable	Basin volume scaled to maintain HRT and depth to area ratio.
DHS	HDPE	kg	Variable	Updated to account for new basin dimensions
Zeolite	Zeolite	kg	Constant	Constant per unit of flow
Zeolite	NaCl (99+%)	kg	Constant	Constant per unit of flow
Zeolite	NaOH	kg	Constant	Constant per unit of flow

Table S7. Scaling approach for AnMBR LCI components

Unit Process	Parts Description	Unit	Constant/ Variable[a]	Scaling Approach
Zeolite	Electricity	kWh	Variable	Scaled according to head associated with modified reaction chamber
Zeolite	Disposal, Brine Injection	m^3	Constant	Constant fraction of flow
UV	Electricity	kWh	Constant	Constant dose rate
UV	Steel	kg	Constant	Number of units increased/decreased to maintain constant UV dose
Chlorination	Concrete	m^3	Variable	Basin volume scaled to maintain HRT and depth to area ratio.
Chlorination	Steel	kg	Variable	Basin volume scaled to maintain HRT and depth to area ratio.
Chlorination	Electricity	kwh	Constant	Constant electricity per unit of flow
Chlorination	Sodium Hypochlorite	kg	Constant	Constant dose rate
Storage	HDPE	kg	Constant	Number of units increased/decreased to maintain constant storage capacity

[a] Constant refers to line items that are constant per unit of flow treated. Examples include chemical dose rates, such as 3 mg of NaOCl per liter of water treated.

212

Table S8. Scaling approach for RVFW LCI components

Unit Process	Parts Description	Unit	Constant/ Variable[a]	Scaling Approach
Fine Screen	Electricity	kWh	Variable	Energy use equation from [40]
Fine Screen	Screening Disposal	kg	Constant	Constant fraction of flow
Fine Screen	Steel	kg	Constant	Constant screen area per unit of flow
Clarifier	Steel	kg	Variable	Basin volume scaled to maintain HRT and depth to area ratio.
Clarifier	Sludge Disposal	m^3	Constant	Constant fraction of flow
Clarifier	Electricity	kWh	Constant	Constant per unit of flow
Equalization	Concrete	m^3	Constant	Basin volume scaled to maintain HRT and depth to area ratio.
Equalization	Steel	kg	Variable	Basin volume scaled to maintain HRT and depth to area ratio.
Equalization	Electricity	kWh	Variable	Pumping energy varied as function of flow, adherence to original design equations

Table S8. Scaling approach for RVFW LCI components

Unit Process	Parts Description	Unit	Constant/ Variable[a]	Scaling Approach
RVFW	Concrete	m^3	Variable	Number of basins varied to maintain constant loading rate
RVFW	Steel - Pumps	kg	Constant	Pump size held constant, number of pumps changed based on flow
RVFW	Steel - Grating	kg	Constant	Number of basins varied to maintain constant loading rate
RVFW	Steel - Rebar	kg	Variable	Number of basins varied to maintain constant loading rate
RVFW	HDPE	kg	Variable	Number of basins varied to maintain constant loading rate
RVFW	Electricity	kwh	Variable	Varied to account for new basin dimensions
RVFW	Lower Media, Crushed Limestone	kg	Variable	Number of basins varied to maintain constant loading rate
RVFW	Middle Media, Gravel	kg	Variable	Number of basins varied to maintain constant loading rate
RVFW	Organic Cover, Wood Chips	kg	Variable	Number of basins varied to maintain constant loading rate
RVFW	Methane	kg	Constant	Constant fraction of flow
RVFW	CO_2, biogenic	kg	Constant	Constant fraction of flow
RVFW	N_2O	kg	Constant	Constant fraction of flow
UV	Electricity	kWh	Constant	Constant dose rate
UV	Steel	kg	Constant	Number of units increased/decreased to maintain constant UV dose
Chlorination	Concrete	m^3	Variable	Basin volume scaled to maintain HRT and depth to area ratio.
Chlorination	Steel	kg	Variable	Basin volume scaled to maintain HRT and depth to area ratio.
Chlorination	Electricity	kwh	Constant	Constant electricity per unit of flow
Chlorination	Sodium Hypochlorite	kg	Constant	Constant dose rate
Storage	Electricity	kWh	Constant	Constant electricity per unit of flow
Storage	HDPE	kg	Constant	Number of units increased/decreased to maintain constant storage capacity

[a] Constant refers to line items that are constant per unit of flow treated. Examples include chemical dose rates, such as 3 mg of NaOCl per liter of water treated.

S1.10 Life cycle inventories

Resulting LCIs for each treatment system are provided in Table S9-S11.

Table S9. Graywater AeMBR LCI.

Unit Process	Inventory Item	Scenario 1<br>Partial Treatment	Scenario 2<br>Full Treatment	Scenario 3<br>Excess Treatment	Units (per m^3 treated graywater)
Centralized Wastewater	Solids and Residual Blackwater	1.40	0.919	0.593	m^3
Potable Water	Avoided	1.00	1.00	0.830	m^3
Fine Screen	Electricity	0.137	0.119	0.107	kWh
	Screening Disposal	4.07E-3	4.07E-3	4.07E-3	kg
	Steel	2.14E-3	1.65E-3	1.34E-3	kg
Equalization	Concrete	1.82E-5	1.62E-5	1.48E-5	m^3
	Steel	1.08E-3	9.64E-4	8.81E-4	kg
	Electricity	0.095	0.095	0.095	kWh
AeMBR	Concrete	2.94E-5	2.59E-5	2.36E-5	m^3
	Steel	1.87E-3	1.63E-3	1.47E-3	kg
	Polyvinyl Fluoride	5.92E-4	5.92E-4	5.92E-4	kg
	Sodium Hypochlorite	7.19E-4	7.19E-4	7.19E-4	kg
	Electricity	0.428	0.428	0.428	kwh
	Methane	4.86E-3	4.86E-3	4.86E-3	kg
	N_2O	5.01E-5	5.01E-5	5.01E-5	kg
	Sludge	8.32E-3	8.32E-3	8.32E-3	m^3
UV	Electricity	0.017	0.017	0.017	kWh
	Steel	3.42E-5	3.42E-5	3.42E-5	kg
Chlorination	Concrete	1.92E-6	1.73E-6	1.59E-6	m^3
	Steel	5.18E-5	4.64E-5	4.26E-5	kg
	Electricity	0.081	0.081	0.081	kwh
	Sodium Hypochlorite	3.20E-3	3.20E-3	3.20E-3	kg NaOCl
Storage	HDPE	7.21E-4	1.11E-3	9.01E-4	kg
Recycled Water Delivery	Electricity	0.100	0.100	0.100	kWh
	PEX pipe, 1/2"	3.66E-4	3.66E-4	3.66E-4	m
	PEX pipe, 1"	2.40E-3	2.40E-3	2.40E-3	m
	PVC pipe, 1"	8.53E-4	8.53E-4	8.53E-4	m
	PVC pipe, 2"	2.79E-4	2.79E-4	2.79E-4	m
Thermal Recovery[a]	Electricity	4.10	4.10	4.10	kWh
	Electricity, Avoided	7.52	7.52	7.52	kWh
	Natural Gas, Avoided	0.901	0.901	0.901	m^3

Unit Process	Inventory Item	Scenario 1<br>Partial Treatment	Scenario 2<br>Full Treatment	Scenario 3<br>Excess Treatment	Units (per m^3 treated graywater)
	R-134a, emission to air	1.56E-5	1.56E-5	1.56E-5	kg

[a] Optional unit process.

Table S10. Mixed Wastewater AeMBR

Unit Process	Inventory Item	Scenario 1<br>Partial Treatment	Scenario 2<br>Full Treatment	Scenario 3<br>Excess Treatment	Units (per m^3 treated wastewater)
Centralized Wastewater	Treatment of Offsite Water	1.40	0.919	0.593	m^3
Potable Water	Avoided	1.00	1.00	0.830	m^3
Fine Screen	Electricity	0.137	0.119	0.107	kWh
	Screening Disposal	9.54E-3	9.54E-3	9.54E-3	kg
	Steel	2.14E-3	1.65E-3	1.34E-3	kg
Equalization	Concrete	1.82E-5	1.62E-5	1.48E-5	m^3
	Steel	1.08E-3	9.64E-4	8.81E-4	kg
	Electricity	0.095	0.095	0.095	kWh
AeMBR	Concrete	2.94E-5	2.59E-5	2.36E-5	m^3
	Steel	1.87E-3	1.63E-3	1.47E-3	kg
	Polyvinyl Fluoride	5.92E-4	5.92E-4	5.92E-4	kg
	Sodium Hypochlorite	7.19E-4	7.19E-4	7.19E-4	kg
	Electricity	0.622	0.622	0.622	kwh
	Methane	5.94E-3	5.94E-3	5.94E-3	kg
	N_2O	2.03E-4	2.03E-4	2.03E-4	kg
	Sludge	0.014	0.014	0.014	m^3
UV	Electricity	0.014	0.014	0.014	kWh
	Steel	3.15E-5	3.15E-5	3.15E-5	kg
Chlorination	Concrete	1.86E-6	1.68E-6	1.55E-6	m^3
	Steel	5.08E-5	4.56E-5	4.19E-5	kg
	Electricity	0.081	0.081	0.081	kwh
	Sodium Hypochlorite	3.60E-3	3.60E-3	3.60E-3	kg NaOCl
Storage	HDPE	7.21E-4	1.11E-3	9.01E-4	kg
Recycled Water Delivery	Electricity	0.100	0.100	0.100	kWh
	PEX pipe, 1/2"	3.66E-4	3.66E-4	3.66E-4	m
	PEX pipe, 1"	2.40E-3	2.40E-3	2.40E-3	m
	PVC pipe, 1"	8.53E-4	8.53E-4	8.53E-4	m
	PVC pipe, 2"	2.79E-4	2.79E-4	2.79E-4	m
Thermal Recovery[a]	Electricity	4.21	4.21	4.21	kWh
	Electricity, Avoided	7.40	7.40	7.40	kWh

Unit Process	Inventory Item	Scenario 1 Partial Treatment	Scenario 2 Full Treatment	Scenario 3 Excess Treatment	Units (per m^3 treated wastewater)
	Natural Gas, Avoided	0.887	0.887	0.887	m^3
	R-134a, emission to air	9.98E-6	1.00	2.00	kg

[a] Optional unit process

Table S11. Graywater AnMBR LCI.

Unit Process	Inventory Item	Scenario 1 Partial Treatment	Scenario 2 Full Treatment	Scenario 3 Excess Treatment	Units (per m^3 treated graywater)
Centralized Wastewater	Treatment of Offsite Water	1.40	0.919	0.593	m^3
Potable Water	Avoided	1.00	1.00	0.830	m^3
Fine Screen	Electricity	0.137	0.119	0.107	kWh
	Screening Disposal	4.07E-3	4.07E-3	4.07E-3	kg
	Steel	2.14E-3	1.65E-3	1.34E-3	kg
Equalization	Concrete	1.82E-5	1.62E-5	1.48E-5	m^3
	Steel	1.08E-3	9.64E-4	8.81E-4	kg
	Electricity	0.095	0.095	0.095	kWh
Chlorination	Concrete	1.92E-6	1.73E-6	1.59E-6	m^3
	Steel	5.18E-5	4.64E-5	4.26E-5	kg
	Electricity	0.081	0.081	0.081	kwh
	Sodium Hypochlorite	5.79E-3	5.79E-3	5.79E-3	kg NaOCl
AnMBR	Concrete	6.53E-5	5.58E-5	4.97E-5	m^3
	Steel	3.56E-3	3.01E-3	2.66E-3	kg
	HDPE	1.56E-4	1.24E-4	1.04E-4	kg
	Polyvinyl Fluoride	1.58E-3	1.58E-3	1.58E-3	kg
	Sodium Hypochlorite	1.92E-3	1.92E-3	1.92E-3	kg
	Electricity	0.726	0.749	0.768	kwh
	Electricity Sensitivity	0.149	0.150	0.152	kwh
	Methane	2.42E-3	2.42E-3	2.42E-3	kg
	Sludge Disposal	7.25E-3	7.25E-3	7.25E-3	m^3
Biogas Recovery	Natural Gas	0.045	0.045	0.045	m^3
DHS	Electricity	0.035	0.035	0.035	kWh
	Methane	1.29E-4	1.29E-4	1.29E-4	kg
	Natural Gas	0.013	0.013	0.013	m^3
	Concrete	3.07E-5	2.75E-5	2.53E-5	m^3
	Steel	1.40E-3	1.28E-3	1.19E-3	kg
	HDPE	3.43E-5	2.76E-5	2.33E-5	kg
Zeolite	Zeolite	0.112	0.112	0.112	kg
	NaCl (99+%)	0.055	0.055	0.055	kg

Table S11. Graywater AnMBR LCI.

Unit Process	Inventory Item	Scenario 1	Scenario 2	Scenario 3	Units (per m^3 treated graywater)
		Partial Treatment	Full Treatment	Excess Treatment	
	NaOH	0.200	0.200	0.200	kg
	Electricity	0.025	0.029	0.034	kWh
	Disposal, Brine Injection	5.51E-3	5.51E-3	5.51E-3	m^3
UV	Electricity	0.017	0.017	0.017	kWh
	Steel	3.42E-5	3.42E-5	3.42E-5	kg
Storage	HDPE	7.21E-4	1.11E-3	9.01E-4	kg
Recycled Water Delivery	Electricity	0.100	0.100	0.083	kWh
	PEX pipe, 1/2"	3.66E-4	3.66E-4	3.66E-4	m
	PEX pipe, 1"	2.40E-3	2.40E-3	2.40E-3	m
	PVC pipe, 1"	8.53E-4	8.53E-4	8.53E-4	m
	PVC pipe, 2"	2.79E-4	2.79E-4	2.79E-4	m

Table S12. Mixed Wastewater AnMBR LCI

Unit Process	Inventory Item	Scenario 1	Scenario 2	Scenario 3	Units (per m^3 treated wastewater)
		Partial Treatment	Full Treatment	Excess Treatment	
Centralized Wastewater	Treatment of Offsite Water	1.40	0.919	0.593	m^3
Potable Water	Avoided	1.00	1.00	0.830	m^3
Fine Screen	Electricity	0.137	0.119	0.107	kWh
	Screening Disposal	9.54E-3	9.54E-3	9.54E-3	kg
	Steel	2.14E-3	1.65E-3	1.34E-3	kg
Equalization	Concrete	1.82E-5	1.62E-5	1.48E-5	m^3
	Steel	1.08E-3	9.64E-4	8.81E-4	kg
	Electricity	0.095	0.095	0.095	kWh
Chlorination	Concrete	1.95E-6	1.75E-6	1.62E-6	m^3
	Steel	5.25E-5	4.71E-5	4.32E-5	kg
	Electricity	0.081	0.081	0.081	kwh
	Sodium Hypochlorite	0.012	0.012	0.012	kg NaOCl
AnMBR	Concrete	6.53E-5	5.58E-5	4.97E-5	m^3
	Steel	3.56E-3	3.01E-3	2.66E-3	kg
	HDPE	2.69E-4	2.12E-4	1.77E-4	kg
	Polyvinyl Fluoride	1.58E-3	1.58E-3	1.58E-3	kg
	Sodium Hypochlorite	1.92E-3	1.92E-3	1.92E-3	kg
	Electricity	0.715	0.737	0.755	kwh
	Electricity Sensitivity	0.148	0.150	0.151	kwh

Table S12. Mixed Wastewater AnMBR LCI

Unit Process	Inventory Item	Scenario 1 Partial Treatment	Scenario 2 Full Treatment	Scenario 3 Excess Treatment	Units (per m^3 treated wastewater)
	Methane	3.49E-3	3.49E-3	3.49E-3	kg
	Sludge Disposal	7.25E-3	7.25E-3	7.25E-3	m^3
	Natural Gas	0.070	0.070	0.070	m^3
DHS	Electricity	0.035	0.035	0.035	kWh
	Methane	1.46E-4	1.46E-4	1.46E-4	kg
	Natural Gas	0.014	0.014	0.014	m^3
	Concrete	3.07E-5	2.75E-5	2.53E-5	m^3
	Steel	1.40E-3	1.28E-3	1.19E-3	kg
	HDPE	6.35E-5	5.16E-5	4.40E-5	kg
Zeolite	Zeolite	0.360	0.360	0.360	kg
	NaCl (99+%)	0.227	0.227	0.227	kg
	NaOH	0.200	0.200	0.200	kg
	Electricity	0.024	0.029	0.034	kWh
	Disposal, Brine Injection	0.023	0.023	0.023	m^3
UV	Electricity	0.034	0.026	0.021	kWh
	Steel	3.15E-5	3.15E-5	3.15E-5	kg
Storage	HDPE	7.21E-4	1.11E-3	9.01E-4	kg
Recycled Water Delivery	Electricity	0.100	0.100	0.083	kWh
	PEX pipe, 1/2"	1.15E-3	1.15E-3	1.15E-3	m
	PEX pipe, 1"	7.56E-4	7.56E-4	7.56E-4	m
	PVC pipe, 1"	2.68E-4	2.68E-4	2.68E-4	m
	PVC pipe, 2"	8.78E-5	8.78E-5	8.78E-5	m

Table S13. Graywater RVFW LCI.

Unit Process	Inventory Item	Scenario 1 Partial Treatment	Scenario 2 Full Treatment	Scenario 3 Excess Treatment	Units (per m^3 treated graywater)
Centralized Wastewater	Treatment of Offsite Water	1.40	0.919	0.593	m^3
Potable Water	Avoided	1.00	1.00	0.830	m^3
Fine Screen	Electricity	0.137	0.119	0.107	kWh
	Screening Disposal	4.08E-3	4.08E-3	4.08E-3	kg
	Steel	2.14E-3	1.65E-3	1.34E-3	kg
Chlorination	Concrete	1.93E-6	1.74E-6	1.60E-6	m^3
	Steel	6.85E-5	5.27E-5	4.28E-5	kg

Unit Process	Inventory Item	Scenario 1<br>Partial Treatment	Scenario 2<br>Full Treatment	Scenario 3<br>Excess Treatment	Units (per m^3 treated graywater)
	Electricity	0.081	0.081	0.081	kwh
	Sodium Hypochlorite	1.50E-3	1.50E-3	1.50E-3	kg NaOCl
RVFW	Concrete	7.45E-5	5.73E-5	9.32E-5	m^3
	Steel	4.99E-5	3.84E-5	6.24E-5	kg
	Steel	7.86E-3	7.86E-3	7.86E-3	kg
	Steel	2.13E-3	1.64E-3	2.67E-3	kg
	HDPE	6.66E-4	5.12E-4	8.32E-4	kg
	Electricity	0.338	0.260	0.423	kwh
	Lower Media, Crushed Limestone	0.017	0.013	0.022	kg
	Middle Media, Gravel	0.061	0.047	0.076	kg
	Organic Cover, Wood Chips	0.065	0.050	0.081	kg
	Methane	7.45E-4	7.45E-4	7.45E-4	kg
	CO_2, biogenic	0.015	0.012	0.019	kg
	N_2O	2.61E-5	2.00E-5	3.26E-5	kg
Clarifier	Steel	6.07E-3	4.67E-3	3.80E-3	kg
	Sludge Disposal	7.32E-3	7.32E-3	7.32E-3	m^3
	Electricity	6.41E-4	6.41E-4	6.41E-4	kWh
Equalization	Concrete	1.74E-5	1.80E-5	1.86E-5	m^3
	Steel	4.98E-4	5.16E-4	5.34E-4	kg
	HPDE	7.23E-5	5.56E-5	7.92E-5	kg
	Electricity	0.197	0.197	0.197	kWh
UV	Electricity	0.056	0.056	0.056	kWh
	Steel	7.88E-5	6.06E-5	4.92E-5	kg
Storage	HDPE	2.16E-3	1.66E-3	1.80E-3	kg
	Electricity	0.045	0.045	0.045	kWh
Recycled Water Delivery	Electricity	0.100	0.100	0.083	kWh
	PEX pipe, 1/2"	3.66E-4	3.66E-4	3.66E-4	m
	PEX pipe, 1"	2.40E-3	2.40E-3	2.40E-3	m
	PVC pipe, 1"	8.53E-4	8.53E-4	8.53E-4	m
	PVC pipe, 2"	2.79E-4	2.79E-4	2.79E-4	m

Table S14. Mixed Wastewater RVFW LCI

Unit Process	Inventory Item	Scenario 1<br>Partial Treatment	Scenario 2<br>Full Treatment	Scenario 3<br>Excess Treatment	Units (per m^3 treated wastewater)
Centralized Wastewater	Treatment of Offsite Water	1.4	0.919	0.593	m^3
Potable Water	Avoided	1	1	0.83	m^3

Unit Process	Inventory Item	Scenario 1	Scenario 2	Scenario 3	Units (per m^3 treated wastewater)
		Partial Treatment	Full Treatment	Excess Treatment	
Fine Screen	Electricity	0.137	0.119	0.107	kWh
	Screening Disposal	9.54E-03	9.54E-03	9.54E-03	kg
	Steel	2.14E-03	1.65E-03	1.34E-03	kg
Chlorination	Concrete	1.92E-06	1.73E-06	1.60E-06	m^3
	Steel	5.15E-05	4.63E-05	4.25E-05	kg
	Electricity	0.081	0.081	0.081	kwh
	Sodium Hypochlorite	1.57E-03	1.57E-03	1.57E-03	kg NaOCl
RVFW	Concrete	7.35E-05	5.65E-05	9.18E-05	m^3
	Steel	1.50E-04	1.15E-04	9.36E-05	kg
	Steel	7.86E-03	7.86E-03	7.86E-03	kg
	Steel	2.10E-03	1.62E-03	2.63E-03	kg
	HDPE	6.66E-04	5.12E-04	8.32E-04	kg
	Electricity	0.338	0.26	0.423	kwh
	Lower Media, Crushed Limestone	0.017	0.013	0.022	kg
	Middle Media, Gravel	0.061	0.047	0.076	kg
	Organic Cover, Wood Chips	0.065	0.05	0.081	kg
	Methane	9.05E-04	9.05E-04	9.05E-04	kg
	CO_2, biogenic	0.015	0.012	0.019	kg
	N_2O	2.61E-05	2.00E-05	3.26E-05	kg
Clarifier	Steel	9.11E-03	7.01E-03	5.69E-03	kg
	Sludge Disposal	0.017	0.017	0.017	m^3
	Electricity	1.50E-03	1.50E-03	1.50E-03	kWh
Equalization	Concrete	1.36E-05	1.40E-05	1.44E-05	m^3
	Steel	3.89E-04	4.00E-04	4.12E-04	kg
	HPDE	7.15E-05	5.50E-05	7.82E-05	kg
	Electricity	0.197	0.197	0.197	kWh
UV	Electricity	0.089	0.068	0.056	kWh
	Steel	7.88E-05	6.06E-05	4.92E-05	kg
Storage	HDPE	2.89E-03	2.77E-03	2.71E-03	kg
	Electricity	0.045	0.045	0.045	kWh
Ozone	Electricity	0.21	0.21	0.21	kWh
	Oxygen	0.131	0.131	0.131	kg
Recycled Water Delivery	Electricity	0.1	0.1	0.083	kWh
	PEX pipe, 1/2"	1.15E-03	1.15E-03	1.15E-03	m
	PEX pipe, 1"	7.56E-04	7.56E-04	7.56E-04	m
	PVC pipe, 1"	2.68E-04	2.68E-04	2.68E-04	m
	PVC pipe, 2"	8.78E-05	8.78E-05	8.78E-05	m

S2. Water Use Scenarios

Indoor flows were defined following [35] using data that reflect the implementation of water conservation efforts typical of new building construction. Residential demand is defined as 35.8 gallons per capita per day (gpcd), less than the national average of 52 gpcd [41]. Commercial demand is defined as 11.3 gpcd following [42]. Graywater generation is assumed to be 72% of residential indoor demand [41] and 37% of commercial indoor demand [43], with the remainder of each flow allocated to blackwater. These assumptions result in the onsite generation of 0.016 million gallons per day (MGD) of graywater or 0.025 MGD of mixed wastewater.

Even with water conservation efforts, non-potable demand, which is defined here as the water required for toilet flushing, laundry and outdoor irrigation, has the potential to vary depending on actual indoor water use efficiency and outdoor irrigation demand. For example, Morelli et al. [35] developed two scenarios to contrast the implementation of high efficiency fixtures and low irrigation demand with average efficiency fixtures and high irrigation demand, with resulting building-wide non-potable demands of 0.0082 MGD and 0.018 MGD, respectively. For this study, an average of the two, or 0.013 MGD, is assumed.

Table S15. Water Use Scenarios (Million Gallons per Day)

Flows within Large Building (1110 Occupants)	Partial Treatment<br>Treatment System Size < Non-potable Demand	Full Treatment<br>Treatment System Size = Non-potable Demand	Excess Treatment<br>Treatment System Size > Non-potable Demand
Non-potable Demand[a]	0.013	0.013	0.013
Graywater Generation[b]	0.016	0.016	0.016
Mixed Wastewater Generation[b]	0.025	0.025	0.025
Treatment System Size[c]	0.010	0.013	0.016
Potable Offset[d]	0.010	0.013	0.013

[a] Average of high reuse and low reuse scenarios described in [35]
[b] [35]
[c] Treatment system size equal to 80%, 100% and 120% of non-potable demand for Scenarios 1, 2 and 3, respectively
[d] Equivalent to non-potable demand satisfied

S3. QMRA Methods

Details of QMRA methodology including exposure routes, use of reference pathogens and dose-response functions, characterization of pathogen concentrations and pathogen treatment are listed in Sections S3.1-S3.4. Section S3.5 lists treatment performance (TP) of specific unit processes for the associated dose.

S3.1 Exposure routes

For toilet flush water and clothes washing, we assumed that 4×10^{-5} L of water was consumed per day for 365 days a year and 10^{-3} L per day for irrigation for 50 days a year, adopted from [44]. We also included accidental ingestion of the treated water for one day of the year for 10% of the population at a volume of 2 L, to be consistent with the exposure assumptions included in the LRT calculation [45].

S3.2 Reference pathogens and dose-response

Of the human-infectious enteric viruses, bacteria and protozoa included in [42], we narrowed the list to the dominant hazards (i.e., *Norovirus* and *Cryptosporidium* spp.). We selected commonly used dose-

response models that relate a healthy adult's dose to a probability of infection based on ingestion (see [45] for more details). For *Norovirus* (doses in genome copies (gc)), two dose-response models were selected to represent the lower- and upper-bounds of predicted risk across the range of available models. The upper-bound, a hypergeometric model for disaggregated viruses [46], predicts relatively high risks among the available models in the relevant dose range. The lower-bound, a fractional Poisson model [47] predicts similar risks as the majority of the published *Norovirus* dose-response models with good empirical fit to the available data (reviewed in Abel et al. [48]). For *Cryptosporidium* spp. (doses in oocysts), we adopted an exponential model based on the U.S. EPA Long Term 2 Enhanced Surface Water Treatment Rule (LT2) Economic Analysis [49] and a fractional Poisson model [47], which results in risks that are much greater than previously predicted in the LT2. LRTs from the guidance document (Table 1) are based primarily on the lower-bound dose-response for *Norovirus* and the upper-bound for *Cryptosporidium* (Sharvelle et al., 2017).

S3.3 Characterization of pathogens in waters

We adopted previously simulated onsite graywater and wastewater pathogen concentrations [50], which used an epidemiology-based approach to describe distributions of pathogen concentrations. The epidemiological approach used data describing population illness rates (as a surrogate for infection) and pathogen shedding characteristics during an infection.

The mixed-use building (with a 1,100-person collection) was modeled using the pathogen concentration simulations from a reference 1,000-person residential building collection system (described in detail in [50]). This simplification was made since most of the collected water in the mixed-use system was from residential use and the difference in population size was small between the reference system and the mixed-use systems.

S3.4 Pathogen treatment

To provide a realistic estimate of risk, we accounted for variability in treatment performance for the MBR and ozone systems, for which pathogen (or surrogate) monitoring data was available (see Tables S16–18 for TP characterizations). Chlorine disinfection performance was set to the LRVs in Table S3 based on the available performance data which showed minimal variation [51,52]. For the RVFW and UV, we did not identify performance data to characterize performance probabilistically; rather, we used the LRVs in Table S3.

The MBR treatment performance was modeled as normal (described in [42]) based on a review of the literature on treatment performance for full scale AeMBR reclaimed water systems between 1992 and 2015 [53]. We did not identify performance data for the AnMBR and assumed that performance was the same. For the ozone treatment performance, we adopted an inverse gaussian characterization based on performance measured over the course of one year at a direct potable reuse plant [51], but we shifted the mean to align with dosing requirements for non-potable treatment (while maintaining the same variance).

Although we did not model UV performance probabilistically, we included a sudden UV treatment failure event, which has been identified previously as a potential problem for finished water quality in potable reuse [51,52]. We modeled a 15-minute UV failure event (UV TP=0) during which poorly treated water mixes with stored, treated water and is consumed over the course of one day. This duration was selected based on previous work [51,52] and assumed that UV treatment failure triggers an alarm and garners a quick response in the form of a manual value close. We modelled the occurrence of a lamp or ballast failure as one event per year [51,54].. For comparison, we also separately modeled risk using the LRVs in Table S3 for indoor use (excluding the irrigation).

S3.5 Treatment Performance

Table S16. Variable Treatment Performance (TP) for Aerobic and Anaerobic MBRs: Mixed Wastewater and Graywater[a]

Unit Process	Virus	Protozoa	Bacteria	Dose	Dose Units
MBR	N(5.6,1) [b]	N(5.0,0.65)	n/a	n/a	n/a
Ozone	n/a	n/a	n/a	n/a	n/a
UV	0	4.0 or 0[c]	n/a	30	mJ/cm^2
Chlorination	4	0	n/a	32	mg-min/L

[a] Source: MBR [53], UV and chlorination [2]

[b] Where N denotes a normal distribution with parameters (mu,sigma)

[c] A LRT of 0 for 15 minutes for 1 day a year due to sudden lamp or ballast failure [51,54]

Table S17. Variable Treatment Performance (TP) for Recirculating Vertical Flow Wetland: Mixed Wastewater[a]

Unit Process	Virus	Protozoa	Bacteria	Dose	Dose Units
RVFW	0.5	1	n/a	n/a	n/a
Ozone	Inverse Gaussian (mu=4.0, lambda= 48.7)	Inverse Gaussian (mu=2.0, lambda= 6.03)	n/a	8.3	mg-min/L
UV	1.0 or 0[b]	4.0 or 0[b]	n/a	55	mJ/cm^2
Chlorination	4	0	n/a	32	mg-min/L

[a] Source: RVFW, UV and Chlorination Guidance [2]

[b]A LRT of 0 for 15 minutes for 1 day a year due to sudden lamp or ballast failure (Pecson et al., 2017, Tng et al., 2015)

Table S18. Variable Treatment Performance (TP) for Recirculating Vertical Flow Wetland: Source-Separated Graywater[a]

Unit Process	Virus	Protozoa	Bacteria	Dose	Dose Units
RVFW	0.5	1	n/a	n/a	n/a
Ozone	n/a	n/a	n/a	n/a	n/a
UV	2.0 or 0[b]	4.0 or 0[b]	n/a	95	mJ/cm^2
Chlorination	4	0	n/a	32	mg-min/L

[a]Source: RVFW, UV and Chlorination [2]

S4. LCCA Methods

Direct cost factors listed in Table S19 were multiplied by unit process costs to estimate the cost of integrating individual treatment processes within the larger wastewater treatment system. Indirect cost

factors listed in Table S20 were multiplied by the sum of unit process and direct costs to estimate the cost of professional services, profit and contingency spending. Table S21 lists the estimated life span of individual system components that determine the time of equipment replacement.

Table S19. Direct Cost Factors

Direct Cost Elements	Direct Cost Factor
Mobilization	0.05
Site Preparation	0.07
Site Electrical	0.15
Yard Piping	0.10
Instrumentation and Control	0.08

Table S20. Indirect Cost Factors

Indirect Cost Elements	Indirect Cost Factor
Miscellaneous Costs	0.05
Legal Costs	0.02
Engineering Design Fee	0.15
Inspection Costs	0.02
Contingency	0.10
Technical Services	0.02
Profit	0.15

Table S21. Estimated Lifespan of System Components

**Unit Process**	**Component**	**Component Lifespan (years)**
Equalization Basin	Basin	40
	Floating Aerator/Mixer	15
Fine Screen	Screen Equipment	15
AeMBR	Basin	40
	Blowers	15
	Diffuser Swing Arm	20
	Diffusers	10
	Membrane	10
	Permeate Pumps	25
	Sludge Pumps	25
AnMBR	Basin	40
	Blower, Biogas Recirculation	15
	Diffuser Swing Arm	20
	Diffusers	10
	Floating Cover	40
	Gas Safety Equipment	15
	Membrane	10
	Mixer	15

Table S21. Estimated Lifespan of System Components

Unit Process	Component	Component Lifespan (years)
	Permeate Pumps	25
	Sludge Pumps	25
	Unit Piping	50
Downflow Hanging Sponge	Blower	15
	Sponge Media	10
	Vessels	40
Zeolite Adsorption System	Feed System	25
	Vessel	40
	Zeolite Regeneration System	15
	Zeolite Replacement System	15
Recirculating Vertical Flow Wetland	Basins	40
	Gravel Media	40
	Piping	50
	Pumps	25
Slant Plate Clarifier	Sludge Pump	25
	Unit	40
UV	Bulb	1
	Quartz Sleeve	5
	Unit	30
Chlorination	Chlorine Pump	25
	Contact Basin	40
Ozone	Contact Basin	40
	Monitoring Equipment	10
	Ozone Generator	10

Equation S1 presents the equation used to estimate interest costs during construction.

$$I_C = \sum (Unit\ Process\ Costs + Direct\ Costs + Indirect\ Costs) \times T_{CP} \times \left(\frac{i_r}{2}\right)$$

Equation S1

Where:
I_C (2016 $) = Interest paid during construction
Unit Process Costs (2016 $) = Total unit process equipment and installation cost
Direct Costs (2016 $) = Total direct costs
Indirect Costs (2014 $) = Indirect costs, including miscellaneous items, legal costs, engineering design fees, inspection costs, contingency and technical services
T_{CP} = Construction period, 3 years based on CAPDETWorks™ default construction period (Hydromantis, 2014)
i_r = Interest rate during construction, %

S5. LCA Methods

Acidification potential, eutrophication potential, and particulate matter formation potential were assessed using U.S. EPA's Tool for the Reduction and Assessment of Chemical and Environmental Impacts (TRACI) impact assessment method, version 2.1 [55,56]. Results for global warming potential (GWP)

category are characterized using factors reported by the Intergovernmental Panel on Climate Change (IPCC) in 2013 with a 100-year time horizon [57]. Fossil fuel depletion potential (FDP) is based on the heating value of the fossil fuel and according to the ReCiPe impact assessment method [58]. Cumulative energy demand (CED) and water use (WU) are inventory indicators and not representative of potential end impacts. CED assesses non-renewable energy extracted and renewable energy utilized. WU is calculated as an inventory of consumptive freshwater withdrawals which are evaporated, incorporated into products and waste, transferred to different watersheds, or disposed into the sea after usage.

Table S22. LCA Metrics

Impact/Inventory Category	Description	Unit
Acidification Potential (AP)	AP quantifies the acidifying effect of substances on their environment. Important emissions leading to terrestrial acidification include sulfur dioxide (SO_2), NOx and ammonia (NH_3). Results are characterized as kg SO_2 eq according to the TRACI impact assessment method.	kg SO_2 eq
Cumulative Energy Demand (CED)	The CED indicator accounts for the total usage of non-renewable fuels (natural gas, petroleum, coal and nuclear) and renewable fuels (such as biomass and hydro). Energy is tracked based on the higher heating value of the fuel utilized from point of extraction, with all energy values summed together and reported on a megajoule (MJ) basis (Hischier et al. 2010).	MJ
Eutrophication Potential (EP)	EP assesses the potential impacts from excessive loading of macro-nutrients to the environment and eventual deposition in freshwater and marine environments. Impacts were assessed according to the TRACI impact assessment method, which calculates a generic eutrophication potential impact that is not specific to either marine or freshwater environments. Both nitrogen and phosphorous compounds are expressed on an equivalent Nitrogen (N) basis.	kg N eq
Fossil Fuel Depletion Potential (FDP)	FDP captures the consumption of fossil fuels, primarily coal, natural gas and crude oil. All fuels are standardized to kg oil eq based on the heating value of the fossil fuel, according to the ReCiPe impact assessment method.	kg oil eq
Global Warming Potential (GWP)	The GWP impact category represents the heat trapping capacity of GHGs over a 100-year time horizon. All GHGs are characterized as kg carbon dioxide equivalents (CO_2 eq) according to the intergovernmental panel on climate change (IPCC) 2013 5th Assessment Report global warming potentials (IPCC 2013).	kg CO_2 eq
Water Use (WU)	The water use indicator accounts for use of freshwater resources abstracted from surface and groundwaters. Water use is an inventory indicator that does not reflect specifically consumptive uses.	m^3 H2O
Particulate Matter Formation Potential (PMFP)	PMFP results in health impacts such as effects on breathing and respiratory systems, damage to lung tissue and premature death (Goedkoop et al. 2013). Primary pollutants (including PM2.5) and secondary pollutants (e.g., SOx and NOx) leading to PM formation are characterized here as kg PM2.5 eq based on the TRACI impact assessment method.	kg PM2.5 eq

Table S22. LCA Metrics

Impact/Inventory Category	Description	Unit
Smog Formation Potential (SFP)	SFP results determine the formation of reactive substances that cause harm to human health and vegetation. Results are characterized here as kg of ozone (O_3) eq according to the TRACI impact assessment method. Some key emissions leading to SFP include CO, methane (CH_4), NOx, NMVOCs and SOx.	kg O_3 eq

S6. Detailed Results

Tables S23 and S24 contain detailed QMRA results listing 95th percentile annual probability of infection for each mixed wastewater and graywater treatment scenario for individual reference pathogens and combined risk.

Table S23. 95th percentile annual probability of infection (ppy) for non-potable reuse including treatment variability and selected failures[a,b]

Reference hazard	Scenario			
	WW MBR	WW Wetland	GW MBR	GW Wetland
1 Cryptosporidium low	8.1E-07	1.2E-04	2.6E-09	1.6E-05
2 Cryptosporidium up	6.6E-06	1.0E-03	2.1E-08	1.2E-04
3 Norovirus low	3.9E-05	4.3E-05	3.0E-07	4.4E-05
4 Norovirus up	2.1E-02	2.4E-02	2.0E-04	2.4E-02
Combined risk low (1,3)	4.2E-05	2.0E-04	3.2E-07	7.0E-05
Combined risk mid-range (2,3)	5.2E-05	1.1E-03	3.8E-07	2.0E-04
Combined risk up (2,4)	2.1E-02	2.6E-02	2.0E-04	2.4E-02

[a]. Assumed 4×10^{-5} L of water consumed per day for 365 days a year; 10^{-3} L of water consumed per day for 50 days a year; and 10% of the population ingesting 2 L per day for 1 day of the year

[b]. For combined risk, numbers in parentheses indicate the pathogen-specific risk used to calculate annual combined risk, using the upper- (up) or lower- (low) bound dose-response

Table S24. 95th percentile annual probability of infection (ppy) for non-potable reuse using LRVs [a,b]

Reference hazard	Scenario			
	WW MBR	WW Wetland	GW MBR	GW Wetland
1 Cryptosporidium low	6.9E-08	7.3E-06	3.5E-10	3.7E-06
2 Cryptosporidium up	5.6E-07	6.0E-05	2.9E-09	3.1E-05
3 Norovirus low	2.2E-05	6.6E-06	8.5E-08	2.8E-05
4 Norovirus up	1.2E-02	4.1E-03	5.5E-05	1.5E-02
Combined risk low (1,3)	2.2E-05	1.4E-05	8.5E-08	3.2E-05
Combined risk mix (2,3)	2.2E-05	6.6E-05	8.8E-08	5.8E-05
Combined risk up (2,4)	1.2E-02	4.2E-03	5.5E-05	1.5E-02

[a] Assumed 4×10^{-5} L of water consumed per day for 365 days a year with 10% of the population ingesting 2 L per day for 1 day of the year

[b]. For combined risk, numbers in parentheses indicate the pathogen-specific risk used to calculate annual combined risk, using the upper- (up) or lower- (low) bound dose-response risk

Tables S25 and S26 list summary LCA results for mixed wastewater and graywater treatment systems, respectively.

Table S25. Summary LCA Results for Mixed Wastewater Treatment Systems

	AeMBR			AnMBR			
**Impact Category**	**No T.R.**	**Electric T.R.**	**Natural Gas T.R.**	**Intermittent**	**Continuous**	**RVFW**	**Units**
Acidification Potential	-5.40E-4	-3.46E-3	9.50E-4	1.88E-3	2.43E-3	-3.30E-4	kg SO_2 eq
Cumulative Energy Demand	-1.80	-32.3	5.41	-4.94	0.743	-0.441	MJ
Eutrophication Potential	4.81E-3	4.64E-3	5.09E-3	5.12E-3	5.17E-3	4.99E-3	kg N eq
Fossil Depletion Potential	-0.039	-0.464	-0.257	-0.098	-0.019	-0.024	kg oil eq
Global Warming Potential	0.054	-1.19	-0.263	0.086	0.321	-0.048	kg CO_2 eq
Particulate Matter Formation Potential	-5.29E-5	-2.40E-4	8.63E-5	7.91E-5	1.20E-4	-2.35E-6	kg $PM_{2.5}$ eq
Smog Formation Potential	2.77E-3	-0.055	0.036	0.079	0.090	6.29E-3	kg O_3 eq
Water Use	-1.19	-1.20	-1.19	-1.19	-1.19	-1.19	m^3 H_2O

Table S26. Summary LCA Results for Graywater Treatment Systems

	AeMBR			AnMBR			
**Impact Category**	**No T.R.**	**Electric T.R.**	**Natural Gas T.R.**	**Intermittent**	**Continuous**	**RVFW**	**Units**
Acidification Potential	-7.30E-4	-3.84E-3	6.30E-4	1.60E-4	7.20E-4	-6.00E-4	kg SO_2 eq
Cumulative Energy Demand	-3.68	-36.3	2.07	-4.84	0.953	-2.84	MJ
Eutrophication Potential	4.72E-3	4.53E-3	4.99E-3	4.88E-3	4.93E-3	4.85E-3	kg N eq
Fossil Depletion Potential	-0.064	-0.518	-0.308	-0.087	-6.03E-3	-0.058	kg oil eq
Global Warming Potential	-0.101	-1.42	-0.480	-0.110	0.129	-0.163	kg CO_2 eq

Table S26. Summary LCA Results for Graywater Treatment Systems

	AeMBR			AnMBR			
**Impact Category**	**No T.R.**	**Electric T.R.**	**Natural Gas T.R.**	**Intermittent**	**Continuous**	**RVFW**	**Units**
Particulate Matter Formation Potential	-6.55E-5	-2.70E-4	6.60E-5	-7.73E-6	2.91E-5	-2.54E-5	kg $PM_{2.5}$ eq
Smog Formation Potential	-9.50E-4	-0.063	0.030	0.022	0.033	1.40E-3	kg O_3 eq
Water Use	-1.19	-1.20	-1.19	-1.19	-1.19	-1.19	m^3 H_2O

Table S27. Summary LCA Results for Graywater Treatment Systems

System Type	Thermal Recovery	Scenario	System Costs over 30 Year Lifespan							
			Electricity	Capital	Materials	Labor	Energy Offset	Centralized Treatment Cost	Avoided Utility Cost	Net NPV
GW AeMBR	None	One	35,161	1,231,889	285,099	1,653,523	-	1,096,516	(1,254,056)	3,048,133
GW AeMBR	None	Two	44,623	1,473,988	305,428	1,767,678	-	936,616	(1,630,273)	2,898,061
GW AeMBR	None	Three	53,988	1,703,919	326,297	1,866,863	-	1,152,759	(1,880,325)	3,223,501
GW AeMBR	Electricity	One	225,754	1,289,927	293,791	1,662,215	(349,321)	1,096,516	(1,254,056)	2,964,827
GW AeMBR	Electricity	Two	292,394	1,549,438	316,727	1,778,977	(454,117)	936,616	(1,630,273)	2,789,763
GW AeMBR	Electricity	Three	358,937	1,796,780	340,204	1,880,770	(558,913)	1,152,759	(1,880,325)	3,090,211
GW AeMBR	Natural Gas	One	225,754	1,289,927	293,791	1,662,215	(123,219)	1,096,516	(1,254,056)	3,190,928
GW AeMBR	Natural Gas	Two	292,394	1,549,438	316,727	1,778,977	(160,185)	936,616	(1,630,273)	3,083,694
GW AeMBR	Natural Gas	Three	358,937	1,796,780	340,204	1,880,770	(197,151)	1,152,759	(1,880,325)	3,451,973
Mixed WW AeMBR	None	One	43,998	832,501	254,483	1,664,916	-	1,096,516	(1,258,578)	2,633,836
Mixed WW AeMBR	None	Two	56,111	953,031	264,279	1,773,414	-	936,616	(1,636,151)	2,347,299
Mixed WW AeMBR	None	Three	68,127	1,061,156	275,014	1,866,740	-	1,152,759	(1,887,560)	2,536,235
Mixed WW AeMBR	Electricity	One	239,598	879,640	261,542	1,671,975	(343,889)	1,096,516	(1,258,578)	2,546,804

Table S27. Summary LCA Results for Graywater Treatment Systems

System Type	Thermal Recovery	Scenario	System Costs over 30 Year Lifespan							
			Electricity	Capital	Materials	Labor	Energy Offset	Centralized Treatment Cost	Avoided Utility Cost	Net NPV
Mixed WW AeMBR	Electricity	Two	310,391	1,014,311	273,456	1,782,591	(447,056)	936,616	(1,636,151)	2,234,158
Mixed WW AeMBR	Electricity	Three	381,087	1,136,578	286,309	1,878,035	(550,223)	1,152,759	(1,887,560)	2,396,984
Mixed WW AeMBR	Natural Gas	One	239,598	879,640	261,542	1,671,975	(121,303)	1,096,516	(1,258,578)	2,769,390
Mixed WW AeMBR	Natural Gas	Two	310,391	1,014,311	273,456	1,782,591	(157,694)	936,616	(1,636,151)	2,523,520
Mixed WW AeMBR	Natural Gas	Three	381,087	1,136,578	286,309	1,878,035	(194,085)	1,152,759	(1,887,560)	2,753,122
GW RVF	None	One	37,507	1,428,279	108,836	1,896,965	-	1,096,516	(1,254,056)	3,314,047
GW RVF	None	Two	42,969	1,751,480	129,927	2,023,241	-	936,616	(1,630,273)	3,253,960
GW RVF	None	Three	64,012	2,077,401	150,930	2,136,575	-	1,152,759	(1,880,325)	3,701,352
Mixed WW RVF	None	One	48,771	1,107,654	63,069	2,305,142	-	1,096,516	(1,258,578)	3,362,575
Mixed WW RVF	None	Two	56,378	1,339,783	70,564	2,421,086	-	936,616	(1,636,151)	3,188,276
Mixed WW RVF	None	Three	79,566	1,565,766	77,708	2,523,578	-	1,152,759	(1,887,560)	3,511,816
GW AnMBR	None	One	51,755	1,737,043	390,436	1,847,523	(7,875)	1,096,516	(1,254,056)	3,861,342
GW AnMBR	None	Two	67,833	2,044,487	425,215	1,983,161	(10,238)	936,616	(1,630,273)	3,816,802

Table S27. Summary LCA Results for Graywater Treatment Systems

System Type	Thermal Recovery	Scenario	System Costs over 30 Year Lifespan							
			Electricity	Capital	Materials	Labor	Energy Offset	Centralized Treatment Cost	Avoided Utility Cost	Net NPV
GW AnMBR	None	Three	84,288	2,335,166	460,110	2,101,111	(12,600)	1,152,759	(1,880,325)	4,240,507
Mixed WW AnMBR	None	One	49,635	1,456,476	384,154	1,823,172	(11,568)	1,096,516	(1,258,578)	3,539,807
Mixed WW AnMBR	None	Two	63,845	1,652,476	416,224	1,949,876	(15,039)	936,616	(1,636,151)	3,367,847
Mixed WW AnMBR	None	Three	78,398	1,830,731	448,415	2,058,725	(18,509)	1,152,759	(1,887,560)	3,662,959
GW AnMBR	None	One	31,896	1,737,043	390,436	1,847,523	(7,875)	1,096,516	(1,254,056)	3,841,482
GW AnMBR	None	Two	40,833	2,044,487	425,215	1,983,161	(10,238)	936,616	(1,630,273)	3,789,802
GW AnMBR	None	Three	49,826	2,335,166	460,110	2,101,111	(12,600)	1,152,759	(1,880,325)	4,206,046
Mixed WW AnMBR	None	One	31,577	1,456,476	384,154	1,823,172	(11,568)	1,096,516	(1,258,578)	3,521,749
Mixed WW AnMBR	None	Two	39,295	1,652,476	416,224	1,949,876	(15,039)	936,616	(1,636,151)	3,343,297
Mixed WW AnMBR	None	Three	47,063	1,830,731	448,415	2,058,725	(18,509)	1,152,759	(1,887,560)	3,631,625

References

1. Bastian, R.; Murray, D. *2012 Guidelines for Water Reuse*; U.S. EPA Office of Research and Development: Washington D.C., 2012;
2. Sharvelle, S.; Ashbolt, N.; Clerico, E.; Hultquist, R.; Leverenz, H.; Olivieri, A. *Risk-Based Framework for the Development of Public Health Guidance for Decentralized Non-Potable Water Systems*; 2017; ISBN 978-1-941242-83-4.
3. Cote, P.; Alam, Z.; Penny, J. Hollow fiber membrane life in membrane bioreactors (MBR). *Desalination* **2012**, *288*, 145–151, doi:10.1016/j.desal.2011.12.026.
4. Suez Z-MOD L Packaged Plants: MBR Platform Systems Featuring LEAPmbr Technology 2017.
5. Yoon, S.-H. *Membrane Bioreactor Processes*; CRC Press: Boca Raton, Florida, 2016;
6. Best, G. Personal Communication Graham Best, GE Power, Water Regional Sales Manager. **2015**.
7. IPCC *IPCC Guidelines for National Greenhouse Gas Inventories*; Intergovernmental Panel on Climate Change, National Greenhouse Gas Inventories Programme: IGES, Japan, 2006; ISBN 4-88788-032-4.
8. Mai, D.T.; Kunacheva, C.; Stuckey, D.C. A review of posttreatment technologies for anaerobic effluents for discharge and recycling of wastewater. *Critical Reviews in Environmental Science and Technology* **2018**, *48*, 167–209, doi:10.1080/10643389.2018.1443667.
9. Boyjoo, Y.; Pareek, V.K.; Ang, M. A review of greywater characteristics and treatment processes. *Water Science and Technology* **2013**, *67*, 1403–1424, doi:10.2166/wst.2013.675.
10. Eriksson, E.; Auffarth, K.; Henze, M.; Ledin, A. Characteristics of grey wastewater. *Urban Water* **2002**, *4*, 85–104, doi:10.1016/S1462-0758(01)00064-4.
11. Ghaitidak, D.M.; Yadav, K.D. Characteristics and treatment of greywater—a review. *Environ Sci Pollut Res* **2013**, *20*, 2795–2809, doi:10.1007/s11356-013-1533-0.
12. Li, F.; Wichmann, K.; Otterpohl, R. Review of the technological approaches for grey water treatment and reuses. *Science of the Total Environment* **2009**, *407*, 3439–3449, doi:10.1016/j.scitotenv.2009.02.004.
13. Metcalf & Eddy; Tchobanoglous, G.; Burton, F. L.; Stensel, H. D.; Tsuchihashi, R. *Wastewater Engineering: Treatment and Reuse*; 5th ed.; McGraw-Hill: Boston, MA, 2014;
14. Feickert, C.A.; Guy, K.; Page, M. *Energy Balance Calculations for an Anaerobic Membrane Bioreactor*; Washington, D.C., 2012; pp. 1–30;.
15. Chang, S. Anaerobic Membrane Bioreactors (AnMBR) for Wastewater Treatment. *Advances in Chemical Engineering and Science* **2014**, *04*, 56–61, doi:10.4236/aces.2014.41008.
16. Lew, B.; Tarre, S.; Beliavski, M.; Dosoretz, C.; Green, M. Anaerobic membrane bioreactor (AnMBR) for domestic wastewater treatment. *Desalination* **2009**, *243*, 251–257, doi:10.1016/j.desal.2008.04.027.
17. Ho, J.; Sung, S. Methanogenic activities in anaerobic membrane bioreactors (AnMBR) treating synthetic municipal wastewater. *Bioresource Technology* **2010**, *101*, 2191–2196, doi:10.1016/j.biortech.2009.11.042.
18. Ho, J.; Sung, S. Anaerobic Membrane Bioreactor Treatment of Synthetic Municipal Wastewater at Ambient Temperature. *Water Environment Research* **2009**, *81*, 922–928, doi:10.2175/106143009X407339.
19. Martinez-Sosa, D.; Helmreich, B.; Netter, T.; Paris, S.; Bischof, F.; Horn, H. Anaerobic submerged membrane bioreactor (AnSMBR) for municipal wastewater treatment under mesophilic and psychrophilic temperature conditions. *Bioresource Technology* **2011**, *102*, 10377–10385, doi:10.1016/j.biortech.2011.09.012.
20. UNFCCC *Clean Development Mechanism: Methodological Tool, Project and leakage emissions from anaerobic digestion*; CDM Methodology; 2012; p. 11;.

21. Christian, S.; Grant, S.; Wilso, D.; McCarthy, P.; Mills, D.; Kolakowski, M. The first two years of full-scale anaerobic membrane bioreactor operation treating high strength wastewater at Kens Foods Inc. *WEF Conference Proceedings* **2010**, 4019–4033.
22. Matsuura, N.; Hatamoto, M.; Sumino, H.; Syutsubo, K.; Yamaguchi, T.; Ohashi, A. Recovery and biological oxidation of dissolved methane in effluent from UASB treatment of municipal sewage using a two-stage closed downflow hanging sponge system. *Journal of Environmental Management* **2015**, *151*, 200–209, doi:10.1016/j.jenvman.2014.12.026.
23. Deng, Q.; Dhar, B.R.; Elbeshbishy, E.; Lee, H.S. Ammonium nitrogen removal from the permeates of anaerobic membrane bioreactors: Economic regeneration of exhausted zeolite. *Environmental Technology (United Kingdom)* **2014**, *35*, 2008–2017, doi:10.1080/09593330.2014.889759.
24. Gross, A.; Shmueli, O.; Ronen, Z.; Raveh, E. Recycled vertical flow constructed wetland (RVFCW)— a novel method of recycling greywater for irrigation in small communities and households. *Chemosphere* **2007**, *66*, 916–923.
25. Sklarz, M.Y.; Gross, A.; Soares, M.I.M.; Yakirevich, A. Mathematical model for analysis of recirculating vertical flow constructed wetlands. *Water Research* **2010**, *44*, doi:10.1016/j.watres.2009.12.011.
26. Alfiya, Y.; Gross, A.; Sklarz, M.; Friedler, E. Reliability of on-site greywater treatment systems in Mediterranean and arid environments - A case study. *Water Science and Technology* **2013**, *67*, 1389–1395, doi:10.2166/wst.2013.687.
27. Gross, A.; Kaplan, D.; Baker, K. Removal of chemical and microbiological contaminants from domestic greywater using a recycled vertical flow bioreactor (RVFB). *Ecological Engineering* **2007**, *32*, 107–114, doi:10.1016/j.ecoleng.2007.06.006.
28. Teiter, S.; Mander, U. Emission of greenhouse gases from constructed wetlands for wastewater treatment and from riparian buffer zones. *Ecological Engineering2* **2005**, *25*, 528–541, doi:10.1016/j.ecoleng.2005.07.011.
29. Atlantic UV Corp. Sanitron® Ultraviolet Water Purifiers. **2007**, 1–10.
30. Primozone® Primozone® GM1-GM2-GM3 - high concentration ozone generators with compact design. **2014**, 1–2.
31. Lenntech Ozone decomposition 2018.
32. Kahraman, A.; Çelebi, A. Investigation of the performance of a heat pump using waste water as a heat source. *Energies* **2009**, *2*, 697–713, doi:10.3390/en20300697.
33. Cipolla, S.S.; Maglionico, M. Heat recovery from urban wastewater: Analysis of the variability of flow rate and temperature in the sewer of Bologna, Italy. *Energy Procedia* **2014**, *45*, 288–297, doi:10.1016/j.egypro.2014.01.031.
34. Greening, B.; Azapagic, A. Domestic heat pumps: Life cycle environmental impacts and potential implications for the UK. *Energy* **2012**, *39*, 205–217, doi:10.1016/j.energy.2012.01.028.
35. Morelli, B.; Cashman, S.; Ma, Cissy; Garland, Jay; Bless, Diana; Jahne, Michael *Life Cycle Assessment and Cost Analysis of Distributed Mixed Wastewater and Graywater Treatment for Water Recycling in the Context of an Urban Case Study*; U.S. Environmental Protection Agency: Cincinnati, OH, 2019; p. 162;.
36. Cashman, S.; Gaglione, A.; Mosley, J.; Weiss, L.; Ashbolt, N.; Hawkins, T.; Cashdollar, J.; Xue, X.; Ma, C.; Arden, S. *Environmental and cost life cycle assessment of disinfection options for municipal drinking water treatment*; Washington, D.C., 2014;
37. Xue, X.; Cashman, S.; Gaglione, A.; Mosley, J.; Weiss, L.; Ma, X.C.; Cashdollar, J.; Garland, J. Holistic analysis of urban water systems in the Greater Cincinnati region: (1) life cycle assessment and cost implications. *Water Research X* **2019**, *2*, 100015, doi:10.1016/j.wroa.2018.100015.
38. Hendrickson, T.P.; Nguyen, M.T.; Sukardi, M.; Miot, A.; Horvath, A.; Nelson, K.L. Life-Cycle Energy Use and Greenhouse Gas Emissions of a Building-Scale Wastewater Treatment and Nonpotable Reuse System. *Environ. Sci. Technol.* **2015**, *49*, 10303–10311, doi:10.1021/acs.est.5b01677.

39. Beveridge, J. Domestic Water System Design for High-rise Buildings. *Plumbing Systems & Design* **2007**, 40–45.
40. Harris, R.W.; Cullinane, M.J.; Sun, P.T. *Process Design and Cost Estimating Algorithms for the Computer Assisted Procedure for Design and Evaluation of Wastewater Treatment Systems (CAPDET)*; U.S. Environmental Protection Agency: Washington, D.C., 1982; p. 1700;.
41. DeOreo, W.B.; Mayer, P.W.; Dziegielewski, B.; Kiefer, J. *Residential End Uses of Water, Version 2*; Denver, CO, 2016;
42. Schoen, M.E.; Jahne, M.A.; Garland, J. Human health impact of non-potable reuse of distributed wastewater and greywater treated by membrane bioreactors. *Microbial Risk Analysis* **2018**, *9*, 72–81, doi:10.1016/j.mran.2018.01.003.
43. Dziegielewski, B.; Kiefer, J.C.; Opitz, E.M.; Porter, G.A.; Lantz, G.L.; DeOreo, W.B.; Mayer, P.W.; Nelson, J.O. *Commercial and Institutional End Uses of Water*; 2000; pp. 298–298;.
44. NRMMC; EPHC; AHMC *Australian Guidelines for Water Recycling: Managing Health and Environmental Risk (Phase 1)*; Natural Resource Management Ministerial Council, Environmental Protection and Heritage Council, Australian Health Ministers' Conference, 2006;
45. Schoen, M.E.; Ashbolt, N.J.; Jahne, M.A.; Garland, J. Risk-based enteric pathogen reduction targets for non-potable and direct potable use of roof runoff, stormwater, and greywater. *Microbial Risk Analysis* **2017**, *5*, 32–43, doi:10.1016/j.mran.2017.01.002.
46. Teunis, P.F.M.; Moe, C.L.; Liu, P.; Miller, S.E.; Lindesmith, L.; Baric, R.S.; Pendu, J.L.; Calderon, R.L. Norwalk virus: How infectious is it? *Journal of Medical Virology* **2008**, *80*, 1468–1476, doi:10.1002/jmv.21237.
47. Messner, M.J.; Berger, P. Cryptosporidium Infection Risk: Results of New Dose-Response Modeling. *Risk Analysis* **2016**, *36*, 1969–1982, doi:10.1111/risa.12541.
48. Abel, N.V.; Schoen, M.E.; Kissel, J.C.; Meschke, J.S. Comparison of Risk Predicted by Multiple Norovirus Dose–Response Models and Implications for Quantitative Microbial Risk Assessment. *Risk Analysis* **2017**, *37*, 245–264, doi:10.1111/risa.12616.
49. U.S. EPA *Occurrence and Exposure Assessment for the Final Long Term 2 Enhanced Surface Water Treatment Rule*; U.S. Environmental Protection Agency: Washington, D.C., 2005;
50. Jahne, M.A.; Schoen, M.E.; Garland, J.L.; Ashbolt, N.J. Simulation of enteric pathogen concentrations in locally-collected greywater and wastewater for microbial risk assessments. *Microbial Risk Analysis* **2017**, *5*, 44–52, doi:10.1016/j.mran.2016.11.001.
51. Pecson, B.M.; Triolo, S.C.; Olivieri, S.; Chen, E.C.; Pisarenko, A.N.; Yang, C.-C.; Olivieri, A.; Haas, C.N.; Trussell, R.S.; Trussell, R.R. Reliability of pathogen control in direct potable reuse: Performance evaluation and QMRA of a full-scale 1 MGD advanced treatment train. *Water Research* **2017**, *122*, 258–268, doi:10.1016/j.watres.2017.06.014.
52. Soller, J.A.; Parker, A.M.; Salveson, A. Public Health Implications of Short Duration, Off-Specification Conditions at Potable Reuse Water Treatment Facilities. *Environ. Sci. Technol. Lett.* **2018**, *5*, 675–680, doi:10.1021/acs.estlett.8b00470.
53. Branch, A. Validation of membrane bioreactors for water recycling 2016.
54. Tng, K., H.; Currie, J.; Roberts, C.; Koh, S.H.; Audley, M.; Leslie, G.L. *Resilience of Advanced Water Treatment Plants for Potable Reuse*; 2015;
55. Bare, J. TRACI 2.0: The Tool for the Reduction and Assessment of Chemical and other Environmental Impacts. *Clean Technologies and Environmental Policy* **2011**, *13*, 687–696, doi:10.1007/s10098-010-0338-9.
56. Bare, J.; Norris, G.A.; Pennington, D.W.; McKone, T. TRACI: The Tool for the Reduction and Assessment of Chemical and other Environmental Impacts. *Journal of Industrial Ecology* **2002**, *6*, 49–78, doi:10.1162/108819802766269539.

57. IPCC *Climate Change 2013: The Physical Science Basis. Contribution of Working Group I to the Fifth Assessment Report of the Intergovernmental Panel on Climate Change*; Cambridge University Press: Cambridge, UK and New York, NY, 2013;
58. Huijbregts, M.A.J.; Steinmann, Z.J.N.; Elshout, P.M.F.; Stam, G.; Verones, F.; Vieira, M.; Zijp, M.; Hollander, A.; van Zelm, R. ReCiPe2016: a harmonised life cycle impact assessment method at midpoint and endpoint level. *Int J Life Cycle Assess* **2017**, *22*, 138–147, doi:10.1007/s11367-016-1246-y.
